# Ethnobotanical study of endemic and non-endemic medicinal plants used by indigenous people in environs of Gullele botanical garden Addis Ababa, central Ethiopia: A major focus on Asteraceae family

**DOI:** 10.3389/fphar.2022.1020097

**Published:** 2022-11-23

**Authors:** Melaku Masresha Woldeamanuel, Mohammed Kasso Geda, Shibani Mohapatra, Tapan Kumar Bastia, Prasanta Rath, Alok Kumar Panda

**Affiliations:** ^1^ Environmental Science Laboratory School of Applied Sciences, Kalinga Institute of Industrial Technology, Deemed to be University, Bhubaneswar, India; ^2^ College of Natural and Computational Sciences, Dire Dawa University, Dire Dawa, Ethiopia

**Keywords:** Ethiopia, ethnobotany, endemism, indigenous botanical knowledge, medicinal plants, Gullele botanical garden, Addis Ababa

## Abstract

Gullele Botanical Garden (GBG) in Addis Ababa, Ethiopia is a joint venture of Addis Ababa government and the university. The garden has been built mainly to conserve the endemic plants and to advance the research on the endemic and non-endemic plants collected from different part of Ethiopia. Many traditional healers from the environs of GBG and different subcities of Addis Ababa depend on the garden for their practice of traditional medicine but there is no systematic documentation of the traditional medicinal knowledge of these healers. The main objective of the present study is to comprehensively document the ethnobotanical and ethnomedicinal information from the traditional healers of different ethnic and cultural groups depending on GBG and to create a database of the endemic plants used by these healers. The ethnobotanical and ethnomedicinal data obtained from 60 traditional healers have been analyzed both qualitatively and quantitatively. A total of 81 medicinal plants belonging to 47 families have been identified. Majority of the plants used belonged to Asteraceae (12) family. The most frequently used plant form and plant parts are herbs and leaves. The major method adopted by the healers for preparation and administration of traditional medicine is crushing and topical, respectively. Skin and general diseases are the most important ailments treated by the healers. The three most cited plants used to treat diseases are *Echinops kebericho* Mesfin (60), *Hagenia abyssinica* (Bruce) J.F.Gmel (60) and Laggera tomentosa (A.Rich.) Sch.Bip. ex Oliv. & Hiern (58). The present study is the first systematic, qualitative, and quantitative ethnobotanical analysis and documentation done on the use of the medicinal plants from GBG for traditional medicine. In addition, our study reveals that *E. kebericho* is endemic and endangered plant and is highly used in traditional medicine. Therefore, GBG authorities should take steps for the propagation and restoration of this plant. Further it is suggested that the pharmacological properties of the roots and leaves of *E. kebericho* should be compared to find the possibility of use of leaves in place of roots for the preparation of traditional medicine which would help in conserving this endemic plant of Ethiopia.

## Introduction

In Ethiopia, the use of medicinal plants to cure and heal diseases has been practiced for a long time and traditional medicine is one of the important components of health care (Giday and Teklehaymanot, 2013). The country harbors around 7,000 plants of higher species, among which around 800 plants are being used by traditional healers to treat a variety of diseases (Teklehaymanot, 2009). Traditional medicine is one of the major health care systems used by over 80 percent of the Ethiopian population (Demie et al., 2018). The main reason for the traditional medicine to be popular in the country is due to its low cost and unavailability of good healthcare systems (Feyssa et al., 2011). Lambert et al. reported that almost more that 90 percent of the birth deliveries in Ethiopia are being carried out by the traditional birth attendants, rather than the trained health workers (Lambert, 2006). Similarly, another study showed that over 35 percent of the population relies on the traditional medicine rather than the pharmaceutical drugs due to lack of money (Hanbin and Gao, 2003). In recent times in Ethiopia, in addition to communicable diseases, the burden of non-communicable diseases has also increased significantly (Girum et al., 2020). The burden of these diseases in the rural and urban populations is different that mainly depends on their sociodemographic conditions, lifestyle, health risks, etc (Chen et al., 2019). Although, the mortality rates for communicable diseases such as respiratory infections, AIDS, tuberculosis, measles, malaria, etc are decreasing but that of the non-communicable diseases such as cardiovascular diseases, diabetes etc, have remained stable over the years.

The high cost of the drugs and modern healthcare have led the people of Ethiopia to heavily depend on the age-old traditional medicinal systems. The practise of traditional medicine is carried in many regions of Ethiopia. One of such regions in Ethiopia is Gullele Botanical Garden (GBG) in Addis Ababa. Addis Ababa city is the capital of Ethiopia and is divided into ten subcities. Gullele is a subcity with an area of nearly 30 sq.km and with a population density of nearly 9,500 per sq.km. There are many ethnic groups that resides in Gullele among which the major groups are Amhara, Oromo, Gamo and Guragie. Botanical gardens are one of the key places for the conservation of a variety of plants. It is known that botanical gardens around the world help to conserve ∼41% of the threatened species (Mounce et al., 2017). Gullele Botanical Garden has been recognised in 2010 as a joint venture between the Addis Ababa government and the university. The garden extends over 705 ha and contributes to the sustainable development goals (SDGs) 6, 7, 13 and 15 (Talemos and Birhanu, 2021). In addition, this garden has been accredited by BGCI (Botanic Gardens Conservation International) until the year 2025. The garden constitutes more than 90 percent of socioeconomic and protected forest. The initial assessment of the GBG landscape showed 223 plant species belonging to nearly 66 families which has currently increased to over 1,200 plant species by collection and *in situ* management techniques. This increase in the GBG’s flora can mainly be attributed to the propagation and planting, reforestation, natural regeneration of endemic plants and limited and sustainable use of the medicinal plants by the traditional healers.

GBG provides a greener environment to the Addis Ababa city and provides an ecosystem for carbon storage, habitat conservation and prevention from soil erosion. Most of the Ethiopian population heavily depend on the use of traditional medicine because of its low cost and high cultural acceptance. As per the garden authorities, traditional healers in Ethiopia have been providing several medical services, treatments and remedies for a long time in Ethiopia. Hence, the people around the garden and in Gullele subcity heavily depend on the plant medicines given by the traditional healers living in the garden’s vicinity. Therefore, the garden’s authorities collaborate closely with the traditional healers or traditional physicians to collect, protect and propagate the medicinal plants species in GBG. Inside GBG there is a medicinal garden which contains and propagates the medicinal important plants which are overharvested. The medicinal garden and the forest section of GBG has a total of 166 medicinal plants which are sustainably used by the certified traditional healers around Gullele and Addis Ababa city. For bio-prospecting and optimum use of the medicinal plant’s proper survey and documentation are required (Awas et al., 2010).

A significant number of traditional healers from different ethnic groups with different culture and tradition depend on the medicinal plants of GBG to practise traditional medicine but there is no systematic documentation of this traditional medicinal knowledge used by the healers. Although, GBG is playing a vital role of transferring the traditional medicinal knowledge from one generation to other there are still many instances where the indigenous knowledge that is available among traditional healers is lost or diluted due to word-to-mouth teaching to the next generation of healers. Therefore, GBG has been selected for the current study and to maximize the documentation the help of the GBG authorities have been taken to identify traditional healers from different ethnic groups. The main objective of the present study is to comprehensively document the ethnobotanical and ethnomedicinal information from the traditional healers of different ethnic and cultural groups depending on GBG and to create a database of the endemic plants used by the healers. Thereafter to quantitatively analyze the data obtained with various ethnobotanical indexes and to uncover the plants which are mostly used in the treatment of diseases by the healers. Identifying the endemic plants mostly used by the healers will help the GBG’s authorities to cultivate and propagate the endemic plants with priority over the others. A secondary objective of this study is to identify the important endemic medicinal plants used by the healers as obtained from the quantitative ethnobotanical analysis and use it for bio-prospecting and discovery of new drug leads.

## Materials and methods

### Study area

**FIGURE 1:**
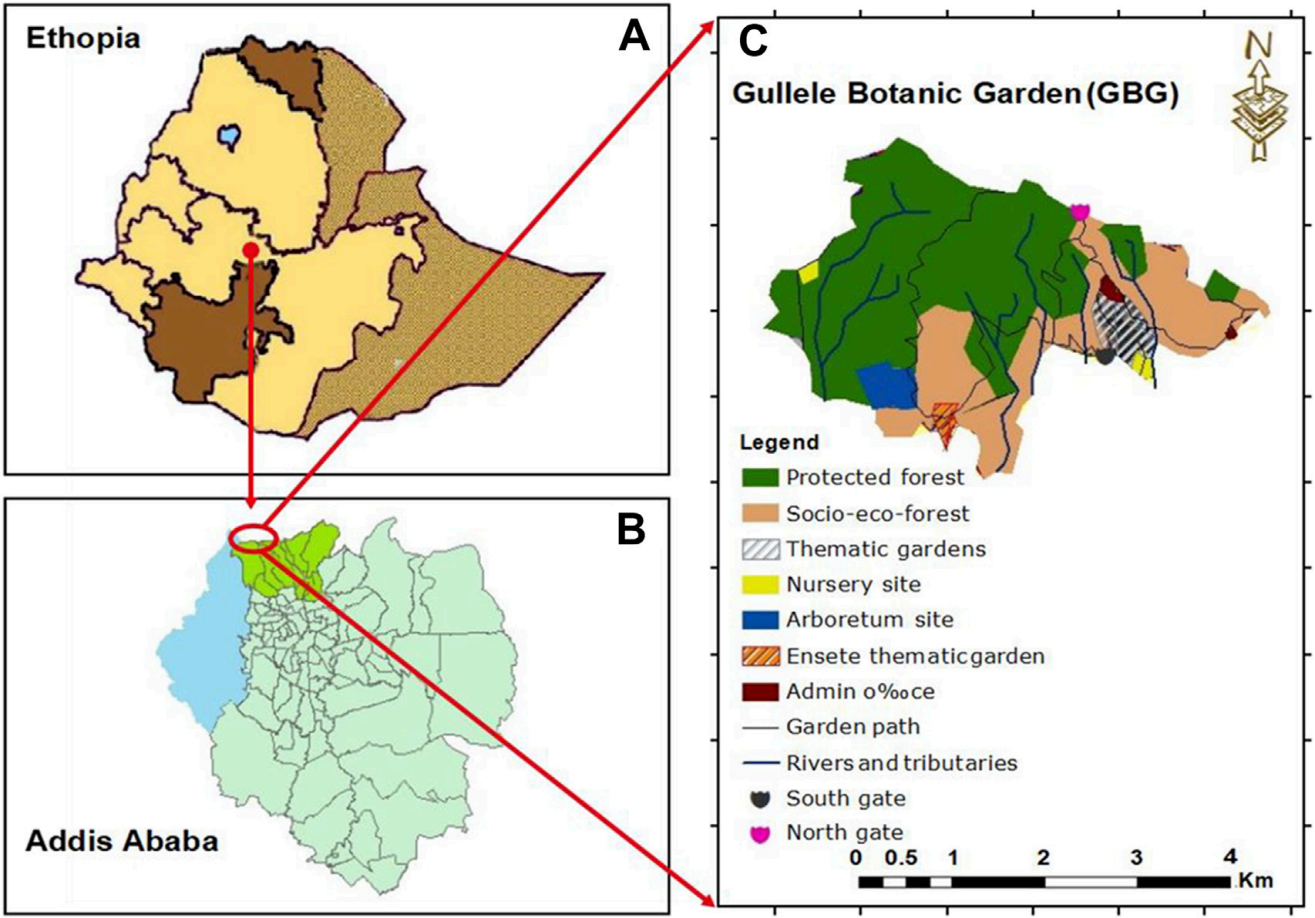
Map of Gullele Botanical Garden. The map shows **(A)** Ethiopia **(B)** the location of Addis Ababa in Ethiopia and **(C)** location of Gullele Botanical Garden and its various areas. Original maps drawn by Fikadu Erenso, Gullele Botanical Garden.

Gullele Botanic Garden is located to the north of Addis Ababa city administration and spans over an area of nearly 705 hectares (Talemos and Birhanu, 2021). It is in the sub cities of Gullele and Kolfe Keraniyo at an altitude of 2,450–2,995 m above the sea level, respectively (Atinafe et al., 2020). The geographical coordinate of the garden is 8°55′ N and 9°05′ N and longitudes 38°05′ E and 39°05′ E (Figure 1). The Entoto mountain adjacent to the garden has an impact on the keeping the climate of this place moderate in nature. The average temperature of the garden is around 15°C–18°C with the average lowest and highest temperature recorded during the year are 7.5°C (in December) and 20.7°C (in February), respectively (Talemos and Birhanu, 2021). The garden receives the maximum precipitation in August, with an annual average rainfall ranging between 1,100 mm and 1,300 mm (Talemos and Birhanu, 2021). The dominant vegetation and the major fauna in this area is *Eucalyptus* globulus and Juniperus, respectively. As this is the first botanical garden in Ethiopia and has a diverse flora and fauna, it is a major destination for eco-tourism and educational outreach. The garden contains around 100 hectares of cultivated plants which harbors nearly 1,600 indigenous plants. Amongst them, around 64 plant species are endemic species, 189 are exotic plants, 900 are indigenous plant species and about 65 are critically endangered species (Woldegerima et al., 2017; Talemos and Birhanu, 2021). The population residing around the garden is from diverse cultural and social background.

### Study sites and interview with informants

The study has been conducted from the month of February 2021 to September 2021. The study sites included both forest and semi-forest vegetation. Nearly 600 people at various work levels work in the garden from the surrounding areas. A total of sixty informants were interviewed who belonged to all kebeles and different traditional medicine practitioners (Supplementary Table S1). Amongst these informants, 40 are male and 20 are female. These informants have been chosen by random and purposive sample approaches according to the guidelines outlined by Martin and his co-workers (Martin, 1995). Each of the informant are administered with a questionnaire and the interviews have been conducted face to face in local languages or in the mother tongue of the informant. Each of the participant were explained the purpose of the study and their consent and approval were taken before the interviews. The ethics code of International Society of Ethnobiology has been followed. To maintain uniformity, the traditional healers or the informants have been chosen from all directions of the garden. All the 60 traditional healers have been chosen based on the feedback of the religious leaders, elders of the villages, Kebele administration and some of the research scholars’ first-hand observations. Demographic information of the informants, i.e. age, gender, educational level, ethnic group and occupation, was recorded. Data was gathered from the informants in the form of semi-structured interviews, group discussions, and guided field walks (Supplementary Figure S2). The main goal behind the interviews was to document the indigenous medicinal knowledge, the medicinal value of the plants and the conservation status of the medicinal plant. During the transect walks, the morphological traits and life forms of the medicinal plants have been noted with the aid of the guide. In addition to this information, the vernacular name of the medicinal plant, the part used for the preparation of the medicine, the method of preparation and the mode of administration and the disease(s) for which the medicinal plant is used was recorded meticulously.

### Ethnobotanical data collection and analysis

#### Plant materials

This study has been conducted following the guidelines for best practices in ethnopharmacological research (Heinrich et al., 2018). During the field studies, around 81 plant specimen samples had been collected. The specimens were numbered on the spot and identified using taxonomic keys from the volumes of Flora of Ethiopia and Eritrea (Hedberg et al., 2009). The visual comparison of the plant specimens had been carried out at the botanical garden of Dire Dawa University. The plant specimens have been deposited at the herbarium of Dire Dawa university (Supplementary Figure S1). Each of the plant species has been further identified by the plant taxonomist at the herbarium of Addis Ababa University and also from Plants of the World Online (https://powo.science.kew.org/).

### Qualitative analysis

The qualitative ethnobotanical data analysis has been done with the aid of Origin Pro 2021 software. To analyze and summarize the data reported on the medicinal plants, parts and life form used, preparation mode and administration mode, descriptive statistics have been used. The therapeutic uses of the medicinal plants collected from the informants in the local languages are translated to English. These diseases, as reported by the informants, are classified into different categories according to the International Classification of Primary Care (Schippers et al., 2010).

### Quantitative analysis

To quantify the collected data, different ethnobotanical indices have been employed. Four ethnobotanical indices have been employed to assess the collected data.

#### Informant consensus factor

Trotter and Logan developed the ICF and readapted by Heinrich to analyze if there is a consensus between the medicinal plants used by the informants and the various ailment categories (Hussain et al., 2019). The informant consensus factor is calculated using the formula

$$ICF=\frac{N_{ur\;}-N_{t\;}}{N_{ur\;}-1}$$

where “N_ur_” is the number of citations for a particular ailment category and “N_t_” is the number of species used for that particular ailment category. The ICF value mainly states the homogeneity present in the ethnobotanical knowledge. A high value of ICF means the informants agree on the use of the medicinal plant species to treat the ailment, while a lower value shows that the informants are secretive about their plant uses or the medicinal plants are chosen at random.

#### Relative frequency of citations

Relative frequency of citation is used to measure the agreement among the traditional healers with the documented plants in a particular study area (Hussain et al., 2019). The relative frequency of citation is given by the formula:

$$Relative\;frequency\;of\;citation\;\left(RFC\right)=\frac{FC}{N}$$

where “FC” is defined as the number of traditional healers that mentioned the use of the plant species and “N” is defined as the total number of informants interviewed.

##### Fidelity level

Fidelity level index is mainly used to find out the most preferred medicinal plant species used to treatany ailment (Hussain et al., 2019). The formulae used for finding Fidelity Level (FL) index are given by:

$$FL=\frac{N_{p}}{N}\times 100$$

where “N_p_” is the number of informants that have cited the medicinal plant used for a particular disease. “N” is the total number of informants citing that particular medicinal plant species used for any diseases.

#### Jaccard’s similarity index

Jaccard’s similarity (JI) index is used to find out the similarity between the operational taxonomic units. It is mainly calculated by comparing the published data from similar taxonomical units by analyzing the medicinal plant species and their uses. The Jaccard’s similarity index is calculated with the aid of the following formulae (Hussain et al., 2019):

$$JI=\frac{C\;}{A+B-C}$$

“A” is the number of medicinal plant species in area “a”, “B” is the number of plant species in area “b” and “C” is the plants species common to area “a” and “b”.

## Results and Discussion

### Demographics of the interviewed informants

The demographic characteristics of the traditional healers have been documented as per the information provided by the healers in face-to-face discussion (Table 1). A total of 60 informants have been interviewed, among which 40 are male and 20 are female traditional health practitioner which gives a male to female ratio of 2:1. Both the male and female traditional healers relied heavily on the local medicinal plant species which they used to treat human diseases. The traditional information of the medicinal plants used by the healers is respected in the community. The traditional healers consider the medicinal knowledge as ancestral and divine and are reluctant to disclose it to the outside world. But on convincing these traditional healers, they shared their knowledge for academic purpose (Supplementary Table S2). Among the traditional healers, ∼68.33 percent, including male and female, are married, ∼25 percent unmarried, and the rest ∼6.67 percent are divorced (Table 1).

**TABLE 1:** Socio-demographical information of the traditional healers.

Categories	Subcategories	Number of informants	Percentage
Age	<25	6	10.00
	25–34	12	20.00
	35–44	9	15.00
	45–54	8	13.33
	55–64	15	25.00
	65–74	6	10.00
	75–84	3	5.00
	85–94	1	1.67
Sex	Male	40	66.7
	Female	20	33.3
Marital Status	Married (M)	29	48.33
	Married (F)	12	20.00
	Unmarried (M)	10	16.67
	Unmarried (F)	5	8.33
	Divorced (M)	1	1.67
	Divorced (F)	3	5.00
Education Level	Modern education	35	58.33
	Religious education	10	16.67
	Uneducated/illiterate	15	25.00

The educational level of the healers as from the collected data has been divided into three categories, i.e., modern education, religious education and uneducated. Most of the informants, i.e., ∼58% are literate and have contemporary education. Ten traditional healers are religiously educated, and they constitute ∼17% of the total informants. The rest 25% did not have any formal education, but the traditional medicinal knowledge has been handed over to them through ancestry (Table 1). In most cases of the healers, the traditional medicinal knowledge about the medicinal plants, methods of preparation, administration, diagnosis of the ailments, and treatment has been passed by word of mouth. Most of the time the traditional medicinal knowledge is passed to the eldest son and daughter of the family, which is mainly based on the character and the ethnobotanical plant knowledge of the eldest.

### Medicinal plant species diversity and endemism

**FIGURE 2:**
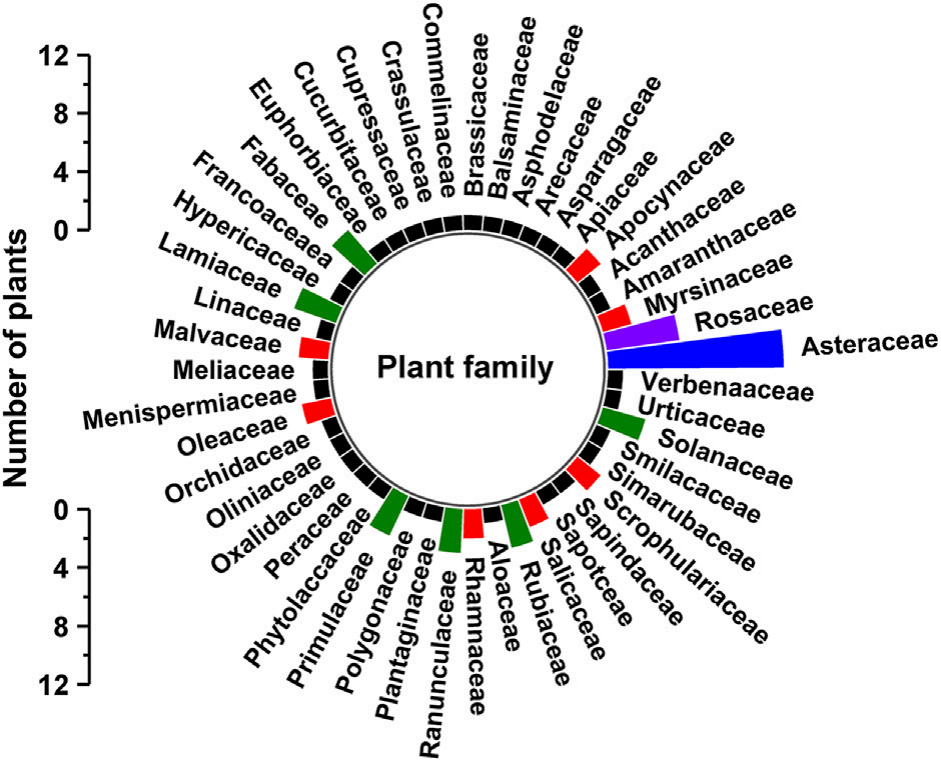
Plant families used in Gullele Botanical Garden. The figure depicts the number of different plant families used by the traditional healers at Gullele botanical for preparing the traditional medicine.

The plants that are used by traditional healers for medicines for human diseases are listed in Table 2 and are arranged in alphabetical order according to their botanical names. Each of the medicinal plant species has been collected in triplicates for voucher specimens and the specimen has been deposited in the Herbarium of Dire Dawa University, Ethiopia. Identification of the species has been done by the experts from Addis Ababa university national herbarium and the scientific names have been crosschecked with the data of Plants of the World Online from Kew website (https://powo.science.kew.org/). The data collected from the field documentation showed that the traditional healers used 81 plant species for which the specimens’ samples have been collected and documented. The 81 medicinal plant species belonged to 47 families (Table 2, Figure 2). In the current study, the number of plants documented is mostly found to be more than the other ethnobotanical studies carried out in Ethiopia (Mesfin et al., 2013; Agisho et al., 2014; Birhanu et al., 2015). The high usage of medicinal plants around Gullele botanical garden mainly indicates the diverse nature of the flora and the rich indigenous knowledge of the healers and the community. It also indicates the natural forest, and the garden has been well preserved by the authorities of the garden. To preserve the garden, the authorities limit access to various parts of the garden, which are only accessible to authorized personnel or traditional healers. The most frequently used plant family cited by the traditional healers is Asteraceae (12), followed by Rosaceae (5) (Table 2, Figure 2). In line with our studies, Chekole and co-workers, in their ethnobotanical study on the environs of Tara-gedam and Amba remnant forests of Libo Kemkem district of Ethiopia, reported the highest number of medicinal plants belonging to the Asteraceae family (Chekole et al., 2015). Similarly, in another ethnobotanical work carried out in Gubalafto district of Ethiopia, most of the plant species used by the traditional healers also belonged to the Asteraceae family (Chekole, 2017). Belayneh et al. from Eastern Ethiopia reported that the traditional medicine used by the people of Harla and Dengego valleys are majorly made from the medicinal plant belonging to the Asteraceae family (Belayneh and Bussa, 2014). In our previous ethnobotanical and ethnomedicinal study on the four districts of eastern India, Asteraceae is among the five major families of medicinal plants employed by traditional healers (Woldeamanel et al., 2022). Similarly, in Bangladesh, Faruque et al. also reported the dominance of medicinal plants from the Asteraceae family in preparing ethnomedicine (Faruque et al., 2018).

**TABLE 2:** Ethnobotanical information of medicinal plants used by traditional healers in Gullele botanical garden in treating human diseases.

Local name	Botanical name	Family	LF	PU	MPAP	CD	Disease indication	RA	Medical uses with ICPC-2	RFC	FL% (ailment)	Voucher specimen no.
									Category			
Sessa	*Alchemilla pedata* Hochst. ex A.Rich.	Rosaceae	H	Wh	Cr	D	Common cold, thyroid, anemia, depression or anxiety	Dr	Digestive	0.16	40.00 (Thyroid)	MDU 146
									Respiratory			
									Endocrine			
Eret	*Aloe percrassa* Tod.	Asphodelaceae	H/S	L	Cr,PW	F	Hemorrhoids, remove dead skin from hair (Dandruff), malaria, eye disease, wound, hair lose	Dr,O,E	General and unspecified	0.20	35.00 (Hemorrhoids)	MDU 111
									Cardiovascular			
									Skin			
									Malaria			
									Eye			
Sariitii	*Asparagus botswanicus* Sebsebe	Asparagaceae	S	R, L	Chw, Pw	F	STDs (L), impotence(L), hemorrhoids (R)	O	General and unspecified,	0.05	28.00 (Impotence)	MDU 154
									Cardio vascular			
									Male genital System			
Azamir	*Bersama abyssinica* Fresen.	Francoaceae	S	L	Cr	F	Diabetes mellitus	O	Endocrine	0.03	70.00 (Diabetes)	MDU199
Yesetan merfe	*Bidens pilosa* L.	Asteraceae	H	L	P	D	Malaria & fungal	O	Malaria	0.08	60.00 (Malaria)	MDU 214
									General			
Sanaafica	*Rhamphospermum nigrum* (L.) Al-Shehbaz	Brassicaceae	H	Se	Pw	F	Gout arthritis	Dr	Endocrine	0.03	50.00 (Gout arthritis)	MDU 187
Qumanya,Tibira	*Brucea antidysenterica* J.F.Mill.	Simaroubaceae	TS	L	P	B	Diarrhoea	O	Digestive	0.20	60.00 (Diarrhoea)	MDU 184
Adaaddo/anfaari	Buddleja polystachya Fresen	Scrophulariaceae	TS	R,L	Pw, Co	F	Evil eye	Ha, Dr	Psychological	0.32	75.00 (Evil eye)	MDU 158
Agam	*Carissa spinarum* L.	Apocynaceae	S	Rb	SmB	F	Evil eye	N	Psychological	0.43	90.00 (Evil Eye)	MDU 108
Zikita	Calpurnia aurea (Aiton) Benth.	Fabaceae	S	L,R	Cr,P(L)	F	Ascariasis (L), rabies (R),	Dr	Endocrine	0.53	50.00 (Ascariasis)	MDU114
							gland cancer (R)		Digestive			
									Skin			
Gura antuta	*Centella asiatica* (L.) Urb.	Apiaceae	H	L	Cr	F	Headache, damaged skin,lose color (vitiligo)	O, N	General and unspecified	0.35	85.00 (Headache)	MDU 149
									Skin			
Yazo egier	*Clematis simensis* Fresen.	Ranunculaceae	Cl	L	Cr	F	Tonsillitis, rheumatic, wounds, bloatsengraving skin with iron	D	Musculoskeletal	0.68	90.00 (Tonsillitis)	MDU 159
									General and unspecified			
									Digestive			
									Skin			
Misrich	*Clerodendrum abilioi* R.Fern.	Lamiaceae	S	R,L	P (L), Pw	B	Gonorrhoea (R), rabies(L), measles(L), tuberculosis (R), colic (L), eye disease (L), malaria(L), swellings in the body(L), wound dressings(L), asthma (R) and anaphrodisiac (R), inducing fertility	N, Dr	General and unspecified	0.73	75.00 (Eye)	MDU 164
									Eye			
									Malaria			
									Respiratory			
									Skin			
									Neurological			
									Child bearing			
									Female and male genital			
Anccote	*Clutia abyssinica* Jaub. & Spach	Peraceae	S	Wh	Cr,Chw	F	Wound	Dr	General and unspecified	0.25	85.00 (wound)	MDU 209
Yehiya anjet	*Commelina benghalensis* L*.*	Commelinaceae	H	St	P	B	Infertility, burns, sore throats, sore eyes, diarrhoea with blood and mucus (dysentery), rashes, and leprosy. ring warm	Dr	Female genital,	0.22	50.00 (Leprosy)	MDU 196
									Skin,			
									Eye,			
									Respiratory			
Not reported	*Erigeron steudelii* (Sch.Bip. ex A.Rich.) Sch.Bip. ex Schweinf.	Asteraceae	H	L	P	F	Wound	Dr	Skin	0.08	75.00 (wound)	MDU 192
Chegogot	*Cyathula uncinulata* (Schrad.) Schinz	Amaranthaceae	H	L	Sq	D	Wound	Dr	Skin	0.66	90.00 (wound)	MDU 118
Astenagirt	*Datura stramonium* L.	Solanaceae	H	L	PW,P		Stomach, intestinal pain, toothache, and fever, scalp, to treat dandruff and falling hair.	O,Dr	General and unspecified	0.75	80.00 (Stomach)	MDU 163
									Digestive			
									Skin			
Not reported	*Delphinium wellbyi* Hemsl.	Ranunculaceae	H	R,Fl	Pw	B	Common cold (Fl),anaemia(R)	Dr, O	General and unspecified	0.06	50.00 (Anaemia)	MDU 110
									Blood			
Sonshi	*Dichrocephala integrifolia* (L.f.) Kuntze	Asteraceae	H	L	Cr	F	Wart	Dr	Skin	0.03	90.00 (wart)	MDU 126
Alimt	*Discopodium penninervium* Hochst.	Solanaceae	S	L	Sq	D	Prolonged labor	Hd	Pregnancy	0.05	80.00 (prolonged labor)	MDU 145
Kitkita	*Dodonaea viscosa* subsp. *angustifolia* (L.f.) J.G.West	Sapindaceae	S	L	Cr,PW	D	Stomach, intestinal pain, toothache, and fever, scalp, dandruff, and falling hair.	O	Digestive	0.75	70.00 (Stomach)	MDU177
									General and unspecified			
Koshommi/anqaqute	*Dovyalis abyssinica* (A.Rich.) Warb.	Salicaceae	S	R,L	Cr	B	Indigestion (R,L) and, increase life expectancy (R,L)	O,Dr	Digestive	0.03	80.00 (Stomach)	MDU 144
									General and unspecified			
Kebericho	*Echinops kebericho* Mesfin	Asteraceae	S	R	Cr,Pw		Migraine, mental illnesss, heart pain, lung TB, leprosy, diarrhoea, kidney, malaria, bilharzias, and amoebic, fever, stomach-ache, and cough, mosquitoes and as a snake repellent, inhaled to fight typhus and fever.	D	Psychological	0.96	100.00 (snake repellent)	MDU 166
									Skin			
									Endocrine			
									Respiratory			
									Digestive			
									General and unspecified			
Sombbo/Duduna	*Ekebergia capensis* Sparrm.	Meliaceae	T	Sb	Co	F	Lung, tuberculosis syphilis & seizure	O	Neurological	0.23	50.00 (lung tuberculosis)	MDU 169
									General and unspecified			
Haanquu	*Embelia schimperi* Vatke	Primulaceae	T,S	L	P	B	Infertility, trachoma	Dr, O	Eye, Female and male gentile	0.30	50.00 (Trachoma)	MDU 176
Barzaafi adi	*Eucalyptus globulus* Labill.	Myrtaceae	T	Fi, Fr	Cr, Pw (Fr)	B	Chronic diarrhoea, headache	O	Digestive	0.95	85.00 (diarrhoea)	MDU 174
									General and unspecified			
Qulqualda	*Eulophia streptopetala* Lindl.	Orchidaceae	H	Wh	P, Smb	F	General weakness (supply energy especially for people living with HIV virus)	O	General and unspecified	0.026	50.00 (weakness)	MDU 189
Ashkita	*Galium abaujense* Borbás	Rubiaceae	H	St,L	P	F	Eczema (chife) (St or L)	Dr	Skin	0.05	100.00 (Eczema)	MDU 152
Heexo	*Hagenia abyssinica* (Bruce) J.F.Gmel.	Rosaceae	T	Fb	Co	B	Tapeworm, fracture making	O	Musculoskeletal	0.17	80.00 (fracture bone)	MDU203
									Digestive			
									Skin			
									General and unspecified			
Hin’dHe,Garam’bba	*Hypericum revolutum* Vahl	Hypericaceae	S	L,Se	Co (Se), In (L)	F	Evil eye, jaundice, & headache	O, N	Psychological. Digestive, General and unspecified	0.15	50.00 (Jaundice)	MDU 200
Arem	*Hypoestes forskaolii* (Vahl) R.Br.	Acanthaceae	H	L	Cr	D	Dandruff	Dr	Skin	0.03	50.00 (Dandruff)	MDU 202
Inshoshilla	*Impatiens rothii* Hook.f.	Balsaminaceae	H	L	Pw	B	Dandruff	HD	Skin	0.05	80.00 (Dandruff	MDU 205
Woynagift	*Pentanema confertiflorum* (A.Rich.) D.Gut.Larr., Santos-Vicente, Anderb., E.Rico & M.M.Mart.Ort.	Asteraceae	H	R	Cr	F	Fungus	Dr	General and unspecified,	0.03	50.00 (Fungus)	MDU 206
									Blood			
Not Reported	*Inula acaulis* Schott & Kotschy ex Boiss.	Asteraceae	H	L	Pw	B	Epistaxis	Dr	Blood	0.01	80.00 (Epistaxis)	MDU 195
Habte harege/Tenbelel	*Jasminum abyssinicum*	Oleaceae	Cl	L	Cr	D	Wound	Dr	Skin, General and unspecified	0.083	90.00 (wound)	MDU173
	Hochst. ex DC.											
Gattiraa,hindhessa	*Juniperus procera*	Cupressaceae	T	R,Sb,Fr	Pw	B	Haematuria (blood in the urine) (R, Sb & Fr)	O	Endocrine,	0.11	75.00 (kidney)	MDU 175
	Hochst. ex Endl.											
Endawula	*Kalanchoe petitiana* A.Rich*.*	Crassulaceae	H	L,Wh.R	Chw,PW	F	Swelling by heating(L), gonorrhoea (Wh), syphilis(R)	O	General and unspecified	0.28	80.00 (Gonorrhoea)	MDU 150
									Skin			
Osote/Gendela	*Lactuca inermis* Forssk.	Asteraceae	H	Wh	Ch	F	Sexual weakness (Impotency)	Dr	Male genital	0.66	90.00 (Impotency)	MDU 148
Kosekoso	Laggera tomentosa (A.Rich.) Sch.Bip. ex Oliv. & Hiern	Asteraceae	H	L	Sq, Pw	F	Tonsil, headache, fumigant in cleaning, leech in livestock	N, Dr	Respiratory	0.68	100.00 (leech)	MDU 127
									General and unspecified			
									Skin			
Talba	*Linum usitatissimum* L.	Linaceae	H	Se	P	F	Wound	Dr	General and unspecified	0.53	90.00 (wound)	MDU 179
Kaseegammojji	*Lippia abyssinica* (Otto & A.Dietr.) Cufod.	Verbenaceae	S	L	P	D	Herpes simplex virus	Dr, O	General and unspecified	0.33	85.00 (herpes)	MDU 140
									Skin			
Wazimma	*Medicago sativa* L.	Fabaceae	H	Fr	Pw	B	Tinea versicolor	O, N	Skin	0.22	50.00 (Tinea versicolor)	MDU 215
Birbira	*Millettia ferruginea* (Hochst.) Hochst. ex Baker	Fabaceae	T	L,Se,Fr,	Cr,Pw,	*D*	Toothaches (L), wound (Se), earache (Fr),insecticidal (Se)	Dr	General and unspecified	0.71	50.00 (wound)	MDU 210
									Skin			
Kataba	*Myrica salicifolia* Hochst. ex A.Rich.	Myricaceae	T	Rb,R	Pw	D	Gonorrhoea (R) and edema leg swelling (Rb)	O	General and unspecified	0.15	65.00 (Gonorrhoea)	MDU 105
Qacamoo	*Myrsine africana* L.	Primulaceae	S	Fr	Cr	B	Dewormers (Kosso)	O	General and unspecified	0.11	85.00 (dewormers)	MDU 147
Shinet	*Myrsine melanophloeos* (L) R.Br. ex Sweet	Primulaceae	T	Lb,Fr	P,Pw	F	Diabetes mellitus (Lb), scabies (Fr)	Dr, O	Endocrine,	0.03	50.00 (Diabetes)	MDU 216
									Skin			
Woyra	*Olea europaea* L.	Oleaceae	T	L	Sq, Co	F	Eye disease & fungus	Dr, E	Eye	0.85	85.00 (Eye)	MDU 142
									General and unspecified			
Tifie	*Olinia rochetiana* A.Juss	Penaeaceae	TS	L	Cr	F	Wound	Dr	General and unspecified	0.06	90.00 (wound)	MDU 151
Tosign	*Origanum vulgare* L.	Lamiaceae	S	L	Cr, PW		Skins sores, aching muscles, asthma; cramping, diarrhoea	Dr,O	Skin	0.86	50.00 (Asthma)	MDU 219
									Respiratory			
									Digestive, musculoskeletal			
Yelmachew	*Oxalis corniculata* L.	Oxalidaceae	H	R,L	P	B	Vomiting (R&L)	O,V	Digestive	0.05	50.00 (Vomiting)	MDU 172
Erbaa	*Phyllopentas schimperi* (Hochst.) Y.D.Zhou & Q.F.Wang	Rubiaceae	S	L	Pw	F	General weakness	O	General and unspecified	0.03	70.00 (weakness)	MDU 194
Qundo arege	*Periploca linearifolia* Quart.-Dill.& A.Rich	Apocynaceae	H	Wh	Cr	B	Heart disease & wound	Dr, O	Cardiovascular	0.18	85.00 (Heart)	MDU 109
Darguu	Phoenix sylvestris (L.) Roxb.	Arecaceae	H	R,L	Cr	F	Diarrhoea with the presence of blood and mucus (L)	V	Blood	0.40	50.00 (wound)	MDU 178
									Digestive,			
							hemorrhage (R), digestive (L)		Skin			
							decreased passing of urine (R)		Cardiovascular			
							facilitate evacuation of the bowels(L)		Urological			
							manage weight(L), asthma(L) and, diarrhoeaL),haemorrhoids(R), Rheumatic pains(R), itches and skin eruption(R,L).		Musculoskeletal			
Handode	*Phytolacca dodecandra* L'Hér.	Phytolaccaceae	S	R,L,Fr	Cr, Pw	B	Benign growth on the skin(L)	O	General and unspecified	0.25	90.00 (Warts)	MDU 119
							(Warts), gonorrhoea (R) and gastritis(Fr)		Digestive			
									Skin			
Ganoxxobbi	*Plantago lanceolata* L.	Plantaginaceae	H	L,St,R	Cr, Pw (L)	B	Gastritis and benign growth on the skin (Wart) (St,R) and trachoma (L)	Dr, O	Eye,	0.32	50.00 (Trachoma)	MDU 182
									Skin, Digestive			
Hoomi,Burayya	*Prunus zippeliana* Miq.	Rosaceae	T	Sb	Cr	D	Sexual weakness (Impotence), tumor (hard swelling)	O	General and unspecified	0.48	65.00 (Impotence)	MDU 181
									Male genital			
Gesho	*Rhamnus* prinoides L'Hér.	Rhamnaceae	TS	Fr	In, P	B	For pigment lost from areas of the skin, causing whitish patches (Vitiligo)	Dr, O	Skin	0.58	70.00 (Vitiligo)	MDU183
Qadiida	*Rhamnus staddo* A.Rich*.*	Rhamnaceae	TS	R	Stb	D	The condition that causes you to wake up during the night to urinate (Nocturia)	O	Psychological, urological	0.18	50.00 (Nocturia)	MDU 155
Qobbo	*Ricinus communis* L.	Euphorbiaceae	H	Fr	Cr	B	Fungal & infection during illegal abortion	Dr	General and unspecified	0.80	60.00 (Fungus)	MDU 220
Incibirri	*Rubia cordifolia* L.	Rubiaceae	Cl	R,L	Co, Pw	B	Hard swelling (Tumor)	Dr	General and unspecified	0.05	50.00 (Tumor)	MDU 221
Amoch	*Rubus steudneri* Schweinf.	Rosaceae	S	L	P, Pw	B	Liver disease (Hepatitis)	O	Endocrine	0.03	60.00 (Hepatitis)	MDU 102
Tsenadam	*Ruta chalepensis* L.	Rutaceae	s	L,FI	Chw,Cr		Frequent, prolonged, and intense crying or fussiness in a healthy infant (Colicky babies) (F), diarrhoea (L), earache(L), heart pain (L), haemorrhoids (FI), influenza symptoms (FI) and intestinal disorders (L)	O	Urological,	080	50.00 (urological)	MDU 243
									Female genital			
									Pregnancy			
Maqimaqqo	*Rumex abyssinicus* Jacq.	Polygonaceae	H	Wh	Co, Cr	B	A small, hard, benign growth on the skin (Wart) and sexual weakness (Impotency)	Dr, O	Skin	0.70	60.00 (Wart)	MDU 112
									Male genital			
Tult	*Rumex nepalensis* Spreng.	Polygonaceae	H	R	Co,Pw	B	A small, hard, benign growth on the skin (Wart) & a dark patch of infected skin (Tinea nigra)	O	Skin	0.13	50.00 (wart)	MDU 113
Haye	*Salix mucronata* Thunb.	Salicaceae	T	Rb	Co, Pw	F	Disease caused of dogs (Rabies)	O	Skin	0.03	60.00 (Rabis)	MDU 106
Hulegeb	*Salvia nilotica* Juss. ex Jacq.	Lamiaceae	H	Wh	Cr	F	Sleep-inducing effect (Sedative), herpes simplex	N, O	Neurological	0.08	50.00 (Herpes)	MDU101
Garda/chifrig	*Sida schimperiana* Hochst. ex A.Rich.	Malvaceae	S	L	P, Ch	F	Wound	Dr	Skin	0.10	50.00 (wound)	MDU 107
Maera	*Spiniluma oxyacantha* (Baill.) Aubrév.	Sapotceae	TS	R	Stb,Sq	B	Wound	Dr	Skin	0.05	60.00 (wound)	MDU 115
Ashkla harege	*Smilax anceps* Willd.	Smilacaceae	LiS	R	P	F	Lung disease (Tuberculosis)	Ha, O	General and unspecified	0.04	50.00 (Tuberculosis)	MDU 122
	*Solanecio gigas* (Vatke) C.Jeffrey	Asteraceae	TS	Wh	P, Pw	B	Sexual weakness (Impotency)	Dr	Male genital	0.03	60.00 (Impotence)	MDU 130
Geber emboye/nechi/	*Solanum marginatum* L.f.	Solanaceae	S	FR,St	Sq	B	Birth control & Sexual, weakness (Impotence) (Fr &St).	O, N	Pregnancy, male genital,	0.88	85.00 (Birth control)	MDU 124
Wulkifa	*Sparrmannia ricinocarpa* (Eckl. & Zeyh.) Kuntze	Malvaceae	H	R	Stb	F	Hepatitis	O	Endocrine	0.11	90.00 (hepatitis)	MDU 123
Enguchite/kalaala	*Stephania abyssinica* (Quart.-Dill. & A.Rich.) Walp.	Menispermiaceae	Cl	Wh	Cr, Pw (R), StB	B	General weakness, heart disease, cholera, skin	Dr, O	General and unspecified	0.65	50.00 (Heart)	MDU 116
									Cardiovascular			
									Digestive			
									Skin			
Yahya shito	*Tagetes minuta* L*.*	Asteraceae	H	L	Ch,p	B	Remedy for colds, respiratory, inflammations, stomach, problem, malaria, anti-parasitic, antiseptic, insecticide and sedative.	O	General and unspecified	0.85	70.00 (Stomach)	MDU 213
									Respiratory			
									Digestive			
									Neurological			
									Skin			
									Musculoskeletal			
Sirabizu	*Thalictrum alpinum* L	Ranunculaceae	H	Wh	Cr, P	B	Stomach pain	N, O	Digestive	0.51	60.00 (Stomach pain)	MDU 156
									Male genital			
Dobbi/samma	*Urtica simensis* Hochst. ex A.Rich.	Urticaceae	H	L	Sq	B	Gastritis & Sexual weakness	O	Digestive	0.33	50.00 (Impotence)	MDU 137
Gurra harre/ketetina	*Verbascum sinaiticum* Benth.	Scrophulariaceae	H	R,L	Pw, In(L,R)	F	TB (R), stomach(l) trouble(L), wound(L), syphilis(R) & heart failure infusion(L)	O, N	General and unspecified	0.40	50., 00 (Digestive)	MDU218
									Cardiovascular			
Dheebicha/girawa	*Gymnanthemum amygdalinum* (Delile) Sch.Bip.	Asteraceae	S	L	Cr, Chw,	F	Toxicity and wound, leech from livestock	Dr, O	General and unspecified	0.91	85.00 (Wound)	MDU 135
Gujo/reji	Orbivestus leopoldii (Sch.Bip. ex Walp.) H.Rob.	Asteraceae	S	R,L	Cr,Co, Chw Pw	F	Snake bite (L) & menstruation problem(R)	Dr, N	Skin	0.75	60.00 (Snake bite)	MDU 136
									Female genital			
Araressa/harege eressa	*Zehneria scabra* subsp. *scabra.*	Cucurbitaceae	Cl	L,St	Stb(R), Co (St)	B	Gout arthritis (L), Wart (St)	Dr,N,O	Skin(St)	0.51	50.00 (Gout arthritis)	MDU197
									Respiratory(L)			

B, Both; Ch, Chewing; Cl, Climber; Co, Concoction; Cr, crushing; Chw, crushing and homogenizing with water; Dr, Dermal; D, dried; Eo, Eye ointment; Fl, Flower; Fb, flower bud; Fr, fruit; F, fresh; Ha, Hanging; Hd, Head; H, herb; In, Infusion; Fi, internal fiber; L, Leaves ; Lb, Leaves bud; Li, Lianas; LF, life form (LF); MPAP, Methods of preparation and application; N, Nasal; O, Oral; PU, Plant part used; P, pounding; Pw, powdering ; RFC, relative frequency of citations; RA, route of administration; R, oot; root bark (Rb); Se, seed; S, shrub; Smb, smoke bath; Sq, squeezing; St, stem; Sb, stem bark; Stb, steam bath; TS, tree/shrub; T, tree; V, vaginal; VN, vernacular name; W, whole.

The frequent use of Asteraceae medicinal plant species in treating human diseases may be attributed to their aromatic compounds and essential oils (Guzel et al., 2015; Fortini et al., 2016). Asteraceae is often considered as one of the largest flowering plant family with a lot of plants having high medical significance (Rolnik and Olas, 2021). The majority of the members of this family have diverse therapeutic applications and have been used in traditional medicine for the past 3,000 years (Nikolić and Stevović, 2015). The Asteraceae family exhibits a wide range of bioactivities such as anti-inflammatory, anti-microbial, antioxidant, anti-parasite, etc. The bioactivities exhibited by the plants belonging to this family are mainly attributed to their bioactive phytochemical compounds, essential oils, saponins, phenolic and polyphenolic compounds, sterols, etc (Koc et al., 2015). Many plants from this family are included in a healthy diet. The roots, leaves, and flowers of this family are rich in vitamins and essential minerals (García-Herrera et al., 2014). They are also used as flavoring agents in food and wines. In the current study, 12 Asteraceae plants has been documented to be used by the traditional healers. Among these Asteraceae plants, phytochemical screening and bioactivities have been performed for many plants. *Bidens pilosa* L. is used by the traditional healers of Gullele Botanical Garden for treating malaria and fungal diseases. This plant has been used in different ethnomedicinal preparations to treat various diseases. The extracts from this plant have shown potent antimicrobial, anti-inflammatory and anti-cancer activity (Bartolome et al., 2013b). These bioactivities can be attributed due to the presence of phenylpropanoids, polyacetylenes, polyphenols, triterpenes, saponins and alkaloids in the plant (Singh et al., 2017). The essential oil from *Bidens pilosa* L. is found to possess a good amount of phenolic compounds with the potential of free radical scavenging activity (Goudoum et al., 2016). Another Asteraceae plant *Dichrocephala integrifolia* (L.f.) Kuntze from our study used by the traditional healers to treat wart, is also found to have anti-cancer, anti-microbial, anti-inflammatory and antioxidant activities (Wabo et al., 2013; Joshi et al., 2020). It is also used as diuretic and to treat eye infections. The extract from this plant exhibits ovicidal and larvicidal activities (Ketcha Wanda et al., 2015). *Dichrocephala integrifolia* (L.f.) Kuntze is also known to exhibit anti-plasmodial and antiprotozoal activities (Mothana et al., 2014). *Tagetes minuta* L. is extensively used as a condiment and herbal tea. In addition, it is also used as a remedy for cold, respiratory diseases, inflammations, sedatives etc. The phytochemical screening of *Tagetes minuta* L. has shown the presence of a diverse array of phytochemical substituents such as alkaloids, flavonoids, steroids, tannins, saponins, glycosides etc (Karimian et al., 2014). It exhibits both antibacterial and antifungal activities (Salehi et al., 2018). It is also seen to exhibit cytotoxic activity against the cancer cell line and hence the natural product from this plant has been considered as a potential target for the for antitumor drugs. *Orbivestus leopoldii* (Sch.Bip. ex Walp.) H.Rob is mainly used by the traditional healers in Gullele Botanical Garden to treat snake bites and menstrual problems. In addition, it is widely used for the treatment of cancer (Tuasha et al., 2022). Recently, a new triterpene isolated from the aerial part of this plant has shown an appreciable cytotoxicity against several cancerous cell lines (Marzouk and Abd Elhalim, 2016). *Laggera tomentosa* (A.Rich.) Sch.Bip. ex Oliv. & Hiern is frequently being used by the healers to treat tonsils and headache in and around Gullele Botanical Garden. The antioxidant and the bactericidal activities of the essential oil of this plant have been documented (Getahun et al., 2020). The dominant compound in the essential oil of this plant are oxygenated monoterpenes and thymol methyl ether (Asfaw et al., 2003). The essential oil from this plant exhibits strong antioxidant and antibacterial activities. In addition to this the leaves of the *Laggera* species have been reported to possess a variety of bioactivities such as antibacterial, anti-inflammatory, antiviral, antioxidant, insecticidal, antifungal, antimycobacterial etc., (Getahun et al., 2019). The other Asteraceae plants in our studies with reported bioactivities are *Lactuca inermis* Forssk and *Solanecio gigas* (Vatke) C.Jeffrey (Janbaz et al., 2013). Both of them are known to have antimicrobial activity against a variety of microbes (Molla Yitayeh and Monie Wassihun, 2022).

In addition to the drug development opportunities that we have discussed for Asteraceae above, a number of plant extracts from this family have been used in the wound healing activity in the rats. The aqueous extract of the aerial part of *Ageratina pichinchensis* (Kunth) R.M. King and H. when applied topically to the incision wound of Sprague-Dawley rats for 8 days, there is an overall healing of the wound by 60% (Romero-Cerecero et al., 2011). Similarly, the topical application of the aqueous and 90% ethanol extract of *Bidens pilosa* L. on the excision wounds of Wistar rats for 9 days at 100 mg/ml, exhibited a wound reduction of nearly 74 percent (Salazar-Gomez and Alonso-Castro, 2022). The ethanol and ethyl acetate fraction of the leaves of *Vernonia scorpioides* (Lam.) Pers. when applied to the excision wound infected with *Staphylococcus aureus* of Wistar rats healed the wound by nearly 40 percent (Salazar-Gomez and Alonso-Castro, 2022). In addition to these preclinical wound healing trials, a clinical study has been done with the hexane-ethyl acetate extract of *Ageratina pichinchensis* (Kunth) R.M. King and H. Rob. The topical application of the extract with carboxymethyl cellulose in a randomized trial in treating the chronic venous leg ulcers of 17 patients showed an ulcer size reduction of around 79 percent in the second month of treatment and it healed completely by the eighth month (Nayak et al., 2009). In a clinical trial, a shampoo formulated from the extracts of *Inula helenium* prevented the loss of hair and stimulated hair growth in patients with androgenetic alopecia (Schreml et al., 2010). These preclinical and clinical studies on Asteraceae plants clearly reveals the potential of this family to contribute towards the better health and treatment of various human diseases.

**FIGURE 3:**
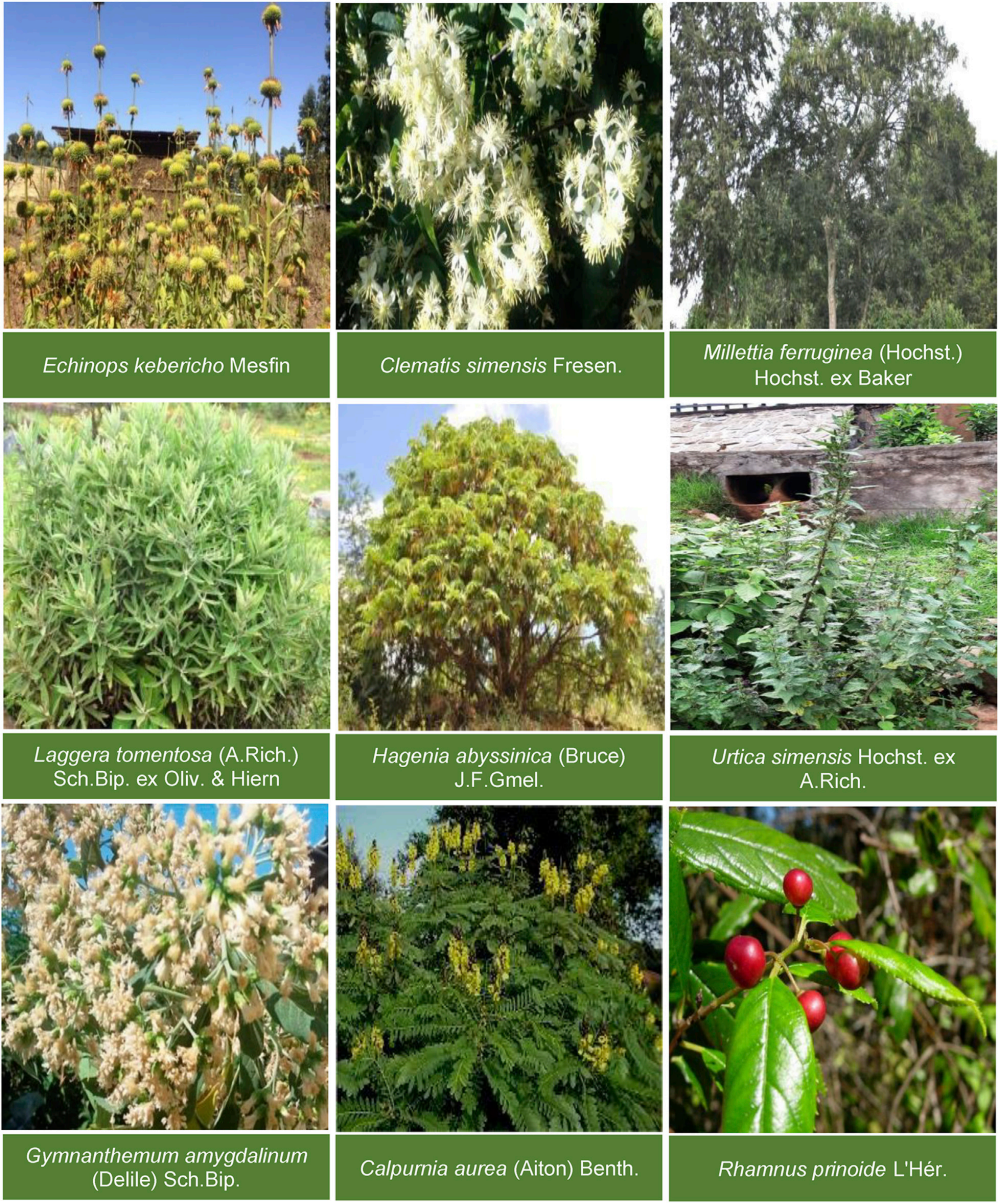
Commonly used medicinal plants. The figure depicts the most commonly and frequently used medicinal plants by the traditional healers of Gullele Botanical Garden.

In addition to the usage of the Asteraceae plants a higher number of medicinal plants from other families in Gullele Botanical Garden reported by the traditional healers in this area shows a diverse flora and a rich knowledge of medicinal plants of the traditional healers. In addition, all the plant species used by the traditional healers are from the garden only and have been used by the healers for a long time. The medicinal plant species *Echinops kebericho* Mesfin (60 citations) has been used to the highest extent by the traditional healers followed by the medicinal plant *Hagenia abyssinica* (Bruce) J.F.Gmel (60 citations) and Laggera tomentosa (A.Rich.) Sch.Bip. ex Oliv. & Hiern (58 citations), respectively (Table 2). The most commonly and frequently used medicinal plants by the traditional healers of Gullele Botanical Garden are shown in Figure 3.

### Endemic flora of the garden

The endemic plants of Ethiopia mainly comprise two listings. One list comprises 476 plant species and the other comprises 480 plant species (Awas, 2009; Ayalew et al., 2022). But considering the International Species Names Index (IPNI) database, the list of endemic plants in Ethiopia has been reduced to 412 medicinal plant species. Endemic plants in Gullele Botanical Garden have been classified into 25 different families. Asteraceae ranks first with 19 plant species, followed by Aloaceae and Fabaceae with seven plant species each (Table 3). The conservation status of each species has been reported in Table 3. In the garden, five plant species are vulnerable (VU), eleven are endangered (EN), twenty one are near threatened (NT), and twenty-seven are least of concern (LC). Among these 64 endemic plants, 20 plants are used as medicinal plants by traditional healers. Hiwot et al. reported that in Ethiopia, there are 44 endemic plants that are medicinally valuable and are used to treat an array of diseases (Ayalew et al., 2022). In this study, there are 20 plants that are medicinally valuable, among which 15 has also been reported in the study done by Hiwot et al. In addition to these 15 plants in the current study more five endemic plants i.e. *Millettia ferruginea* (Hochst.) Hochst ex Baker, *Spiniluma oxyacantha* (Baill.) Aubrév., *Urtica simensis* Hochst. ex A.Rich, *Pentanema confertiflorum* (A.Rich.) D.Gut.Larr., Santos-Vicente, Anderb., E.Rico & M.M.Mart.Ort. and *Kalanchoe petitiana* A. Rich plants have also been found to be used by traditional healers to treat a variety of diseases (Table 2). Among these 20 endemic plants, three plants are endangered or nearly threatened, which must be conserved.

**TABLE 3:** The endemic flora of Gullele Botanical Garden.

Botanical name	Life form	Family	Local name	Medicinal usage	Status of species
*Vachellia negrii* (Pic.Serm.) Kyal. & Boatwr.	Tree	Fabaceae	Bazera Girar	NR	VU
*Vachellia prasinata* (Hunde) Kyal. & Boatwr.	Tree	Fabaceae	Girar	NR	EN
*Acanthus sennii* Chiov.	Shrub	Acanthaceae	Kosshikoshile	NR	LC
*Agrostis diffusa* S.M. Phillips	Grass	Poaceae	Saree	NR	LC
*Alchemilla haumannii* Rothm.	Herb	Rosaceae	Yemider koso	NR	EN
*Aloe ankoberensis* M.G.Gilbert & Sebsebe	Succulent	Asphodelaceae	Erete	R	NT
*Aloe debrana* Chrstian	Succulent	Asphodelaceae	Erate	R	NT
*Aloe kefaensis* M.G.Gilbert & Sebsebe	Succulent	Asphodelaceae	Erate	NR	NT
*Aloe pirottae* A.Berger	Succulent	Asphodelaceae	Erate	NR	NT
*Aloe pulcherrima* M.G.Gilbert & Sebsebe	Succulent	Asphodelaceae	Seate Erate	R	NT
*Aloe sinana* Reynolds	Succulent	Asphodelaceae	Erate	R	NT
*Aloe yavellana* Reynolds	Succulent	Asphodelaceae	Erate	R	NT
*Berkheya chiesiana* Chiov.	Herb	Asteraceae	bloudisseldoring	NR	LC
*Ceropegia aristolochioides* subsp. *aristolochioides*	Herb	Asclepiadaceae	Not reported	NR	NT
*Chiliocephalum schimperi* Benth.	Herb	Asteraceae	Kimo	NR	LC
*Cineraria abyssinica* Sch.Bip. ex A.Rich.	Herb	Asteraceae	Esemerfe	NR	LC
*Conyza flabellata* Mesfin	Shrub	Asteraceae	Hairy fleabane.	NR	NT
*Crotalaria exaltata* Polhill	Shrub	Fabaceae	Abachenane enchete	NR	NT
*Crotalaria rosenii* (Pax) Milne-Redh. ex Polhill	Shrub	Fabaceae	Abachenane enchete	NR	NT
*Crassocephalum macropappus* S.Moore	Herb	Asteraceae	Mandilo/Enset	NR	LC
*Cyanotis polyrrhiza* Hochst. Ex Hassk.	Herb	Commelinaceae	Sokoro	NR	LC
*Echinops ellenbeckii* O. Hoffm.	Shrub	Asteraceae	Not reported	NR	EN
*Echinops kebericho* Mesfin	Shrub	Asteraceae	qarabbicho	R	EN
*Echinops longisetus* A. Rich.	Shrub	Asteraceae	Not reported	R	EN
*Eragrostis tef* (Zuccagni) Trotter	Herb	Poaceae	Teffe	NR	LC
*Erythrina brucei* Schweinf.	Tree	Fabaceae	Koriche	NR	LC
*Euphorbia awashensis* M.G.Gilbert	Shrub	Euphorbiaceae	kulikal	NR	LC
*Euphorbia baleensis* M.G.Gilbert	Shrub	Euphorbiaceae	Anterfa	NR	LC
*Helichrysum harennense Mesfin*	Herb	Asteraceae	Not reported	NR	LC
*Impatiens rothii* Hook.f.	Herb	Balsaminaceae	inshoshila	R	LC
*Impatiens tinctoria* A.Rich.	Herb	Balsaminaceae	Burii/buri jel’dessa	NR	LC
*Pentanema confertiflorum* (A.Rich.) D.Gut.Larr., Santos-Vicente, Anderb., E.Rico & M.M.Mart.Ort.	Shrub	Asteraceae	Not reported	R	NT
*Microcharis cana* (Thulin) Schrire	Herb	Fabaceae	hairy indigo.	NR	LC
*Chrysojasminum stans* (Pax) *Banfi*	Shrub	Oleaceae	Anokitel	NR	VU
*Justicia bizuneshiae* Ensermu	Herb	Acanthaceae	Ensermu	NR	LC
*Kalanchoe petitiana* A. Rich.	Herb	Crassulaceae	Bosoqqee	R	LC
*Kniphofia foliosa* Hochst.	Herb	Asphodelaceae	አበልቢላ/የዝንጀሮ አገዳ	R	VU
*Kniphofia schimperi* Baker	Herb	Asphodelaceae	አበልቢላ/yezijero Ageda	NR	NT
Laggera tomentosa (A.Rich.) Sch.Bip. ex Oliv. & Hiern	Herb	Asteraceae	Koskoso	R	NT
*Leucas stachydiformis* (Benth.) Hochst. ex Briq.	Herb	Lamiaceae	Muka Bofta.	NR	NT
*Lippia abyssinica* (Otto & A.Dietr.) Cufod.	Shrub	Lamiaceae	Kasee gammojii	R	LC
*Lobelia rhynchopetalum* Hemsl.	Shrub	Campanulaceae	Jibera	R	LC
*Gymnosporia addat* Loes.	Tree	Celastraceae	Atate	NR	NT
*Mikaniopsis clematoides* (Sch.Bip. ex A.Rich.) Milne-Redh.	Climber	Asteraceae	Katisa(ኦሮምኛ)	NR	LC
*Millettia ferruginea* (Hochst.) Hochst. ex Baker	Tree	Fabaceae	Birbira	R	EN
*Afroligusticum mattirolii* (Chiov.) P.J.D.Winter	Herb	Apiaceae	sirabizu	NR	VU
*Phagnalon abyssinicum* Sch. Bip. ex A.Rich	Herb	Asteraceae	Nibe asele	NR	NT
*Phragmanthera macrosolen* (Steud. ex A.Rich.) M.G.Gilbert	Parasitic	Loranthaceae	Digalu dhebichaa	R	NT
*Plectocephalus varians* (A.Rich.) C.Jeffrey	Herb	Asteraceae	Eseyohanis (ግዕዝ)	NR	EN
*Searsia glutinosa* (Hochst. ex A.Rich.) Moffett	Shrub	Ananthaceae	xaxxessa	NR	VU
*Clinopodium paradoxum*	Herb	Lamiaceae	Tzatrah	NR	NT
*Senecio steudelii* Sch.Bip. ex A.Rich.	Herb	Asteraceae	Kebekabo (አማርኛ)	NR	NT
*Senecio myriocephalus* Sch. Bip. Ex A.Rich.	Herb	Asteraceae	Sinibita (ጉራጊኛ)	NR	LC
*Senecio schimperi* Sch.Bip. ex Hochst.	Herb	Asteraceae	Tseyaadegi/Tigrega/	NR	NT
*Spiniluma oxyacantha* (Baill.) Aubrév.	Shrub/tr	Sapotaceae	Qontir	R	EN
*Solanecio gigas* (Vatke) C.Jeffrey	Shrubby	Asteraceae	Yeshkoko Gomene	R	LC
*Solanum marginatum* L.f.	Shrub	Solanaceae	Hi’ddi gurguda	R	LC
*Taverniera abyssinica* A. Rich.	Shrub	Fabaceae	Dingetenga	NR	EN
*Thymus schimperi* Ronniger	Herb	Lamiaceae	Tosigne	NR	LC
*Trifolium calocephalum* Fresen.	Herb	Fabaceae	Gosa siidisa	NR	LC
*Wendlandia arabica* Deflers	Shrub	Rubiaceae	Rondeletia heynei,	NR	EN
*Xerophyta rippsteinii* L.B.Sm., J.-P.Lebrun & Stork	Herb	Velloziaceae	bobbejaanstert	NR	LC
*Urtica simensis* Hochst. ex A.Rich.	Herb	Urticaceae	Sama	R	EN
*Orbivestus leopoldii* (Sch.Bip. ex Walp.) H.Rob.	Shrub	Asteraceae	Gujo	R	LC

Key: NR, not reported in the current study; R, reported in Table 2; LC, least of concern; NT, near threatened; VU, vulnerable; EN, endangered.

### Life forms and plant parts

**FIGURE 4:**
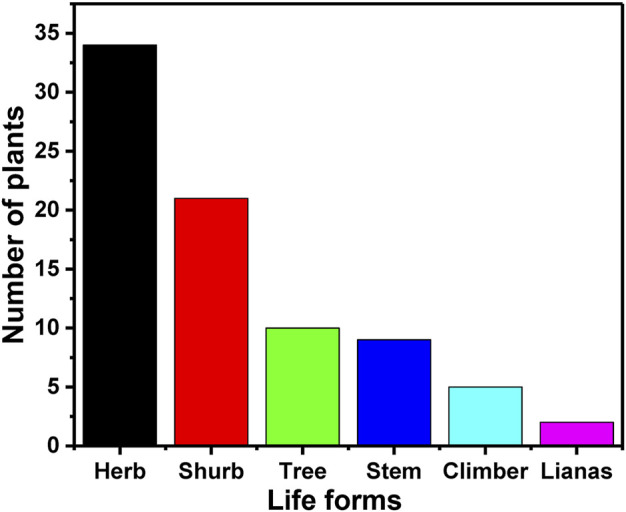
Life forms of the medicinal plants. The figure depicts the number of different plants categorised into different life forms used by the traditional healers at Gullele Botanical Garden for preparing the traditional medicine.

In the present study the analysis of the collected data revealed that the life form which is mostly used by the healers are herbs (∼42%) followed by shrubs (∼26%), trees (∼12%), stem (∼11%), climber (∼6%) and lianas (∼3%) the least used by the traditional healers (Table 2 and Figure 4). Herbs are used by most of the healers because of their ubiquitous nature and easy availability. Besides this, due to the geographical location of Gullele Botanical Garden in Entoto hill, the growth of herbs is more abundant among all the life forms. Healers also prefer herbs because the bioactive fractions of the herbs can be easily extracted and used for herbal formulations (Shrestha and Dhillion, 2003). In addition to herbs, shrubs are also easily available due to which the traditional healers use it frequently. Lulekal et al. also reported the usage of a higher percentage of herbs in an ethnomedicinal study in the Amhara region of Ethiopia (Lulekal et al., 2013). Similarly, Tefera et al. also found a substantial usage of herbs and shrubs in the ethnobotanical study conducted in Hawassa Zuria district of southern Ethiopia (Tefera and Kim, 2019). In another study, Mesfin et al. and Giday et al. reported herbs are the dominant form used by the traditional healers(Mesfin et al., 2009; Giday et al., 2010). Ethnobotanical studies carried out in Wonago district showed a larger use of shrubs in preparing the traditional medicine. The dominant use of herbs by the traditional healers in our current study is in line with the supremacy of herbal species in the traditional medicinal plant portfolios both in Ethiopia and the world (Giday et al., 2003; Tabuti et al., 2003; Muthu et al., 2006; Yineger et al., 2007; Giday et al., 2010).

**FIGURE 5:**
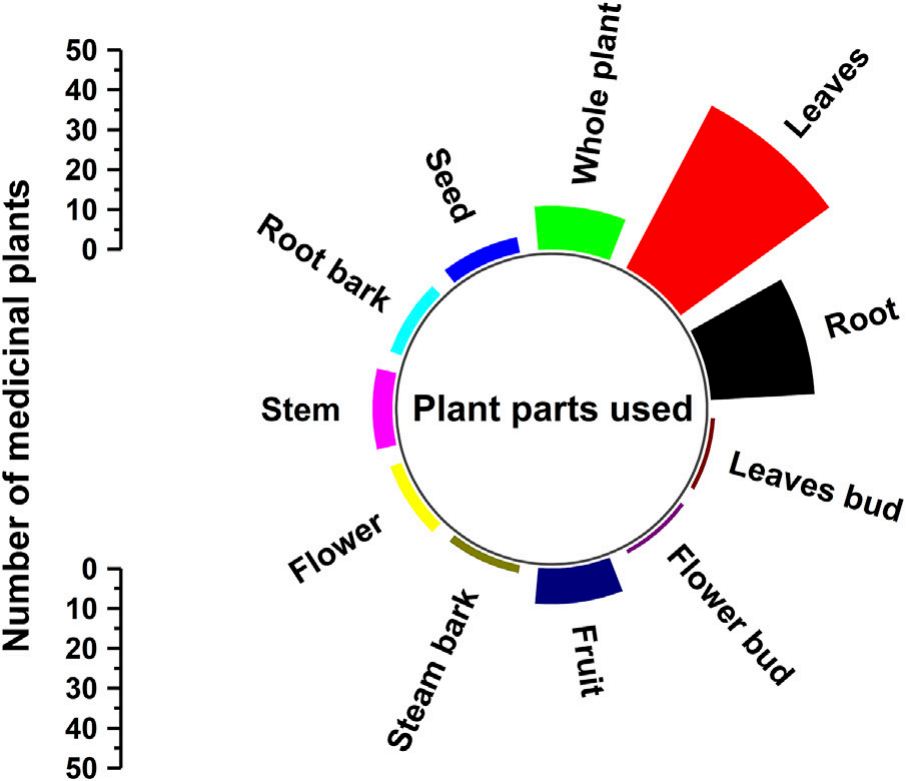
Plant parts used in for traditional medicine. The figure depicts the different part of the plants used by the traditional healers at Gullele Botanical Garden for preparing the traditional medicine and the number of citations.

In the current study, the traditional healers have used different parts of the medicinal plant to prepare the medicines. Among all the plants parts, the most commonly used is leaves (46 citations) followed by roots (26 citations) and whole plant (11 citations) (Table 2 and Figure 5). Besides, the traditional healers have used the seeds, root bark, stem, flower, stem bark, fruits, flower bud and leave buds of the medicinal plants also (Figure 5 and Table 2). The more usage of leaves in herbal formulations may be attributed because of their easiness in extracting phytochemical and their active secondary constituents. The whole plants that the traditional healers use are mostly herbs (Table 2). It is important to note that the usage of medicinal plants’ roots is harmful for the propagation and regeneration of the medicinal plants. Therefore, in Gullele Botanical Garden, both the authorities and traditional healers only use the roots of the medicinal plants after seeds are collected for the plant for the further propagation of the medicinal plant. Therefore, wherever possible the traditional healers use leaves of the medicinal plants rather than the seeds and fruits as it helps in the plant’s conservation. The large diversity of plant part used show the deep-rooted understanding of the traditional healers in using the medicinal plants for the preparation of traditional medicine. Like our results, Belayneh and co-workers reported the dominant use of leaves by the healers of Harla and Dengego valleys of eastern Ethiopia (Belayneh and Bussa, 2014). Similarly, Wondimu et al. also found in their ethnobotanical study the usage of leaves for preparing the medicine to be the highest followed by the roots (Wondimu et al., 2007). In many other ethnobotanical studies conducted in Ethiopia, leaves are mostly used by traditional healers for medicine preparation, as it does not pose a threat to the survival of the medicinal plants (Yineger et al., 2008; Bekalo et al., 2009; Yirga, 2010b). But in a study conducted in Benshangul-Gumuz of Ethiopia, it has been reported that a major part of the plants used for medicinal preparation is the root and bark (Flatie et al., 2009). Similarly, Teklehaymanot et al. in their ethnobotanical study in the Zegie peninsula of Ethiopia found the usage of roots is maximum, followed by leaves (Teklehaymanot and Giday, 2007). It is important to note that medicine prepared from the roots, rhizomes, bulbs, bark and stem pose a serious threat to the survival of the plants.

### Drug preparation methods and administration routes

**FIGURE 6:**
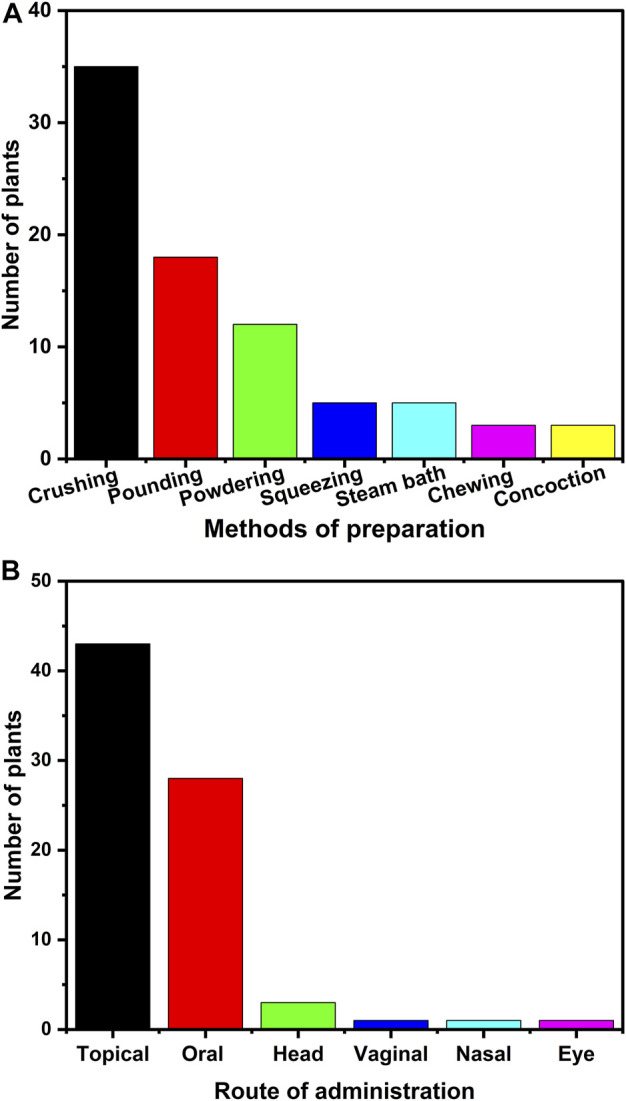
Preparation and administration methods. The figure illustrates the **(A)** methods of preparation adopted by the traditional healers to prepare the medicine **(B)** administration routes of the traditional medicines employed by the traditional healers.

In the present work, the healers have adopted various preparation modes to prepare the medicine, as represented in Figure 6A and Table 2. The common preparation method involved crushing the medicinal plant mostly in its fresh form, with a citation of 35 (Figure 6). Apart from crushing, pounding and powdering, has also been adopted by various traditional healers which have 18 and 12 citations, respectively (Figure 6A). Besides these preparation methods, the medicinal plants are also squeezed and steamed to prepare the traditional medicine (Figure 6A). Apart from using fresh medicinal plants to prepare traditional medicine, dried medicinal plants are also used. In line with our studies, Demie et al. also observed crushing and pounding as a common mode of preparation of traditional medicine from medicinal plants (Demie et al., 2018). Besides this, various other ethnobotanical studies in Ethiopia also noted crushing as the major method adopted for preparing the traditional medicine (Alemayehu et al., 2015; Demie et al., 2018). The data from our study also revealed that most of the preparation of the remedies involves a single medicinal plant (Table 2). In agreement with our results, Asase et al. and Chekole et al. also found a high incidence of remedy preparation from single medicinal plant from their ethnobotanical investigation in Ghana and Ethiopia, respectively (Asase et al., 2010; Chekole et al., 2015).

**FIGURE 7:**
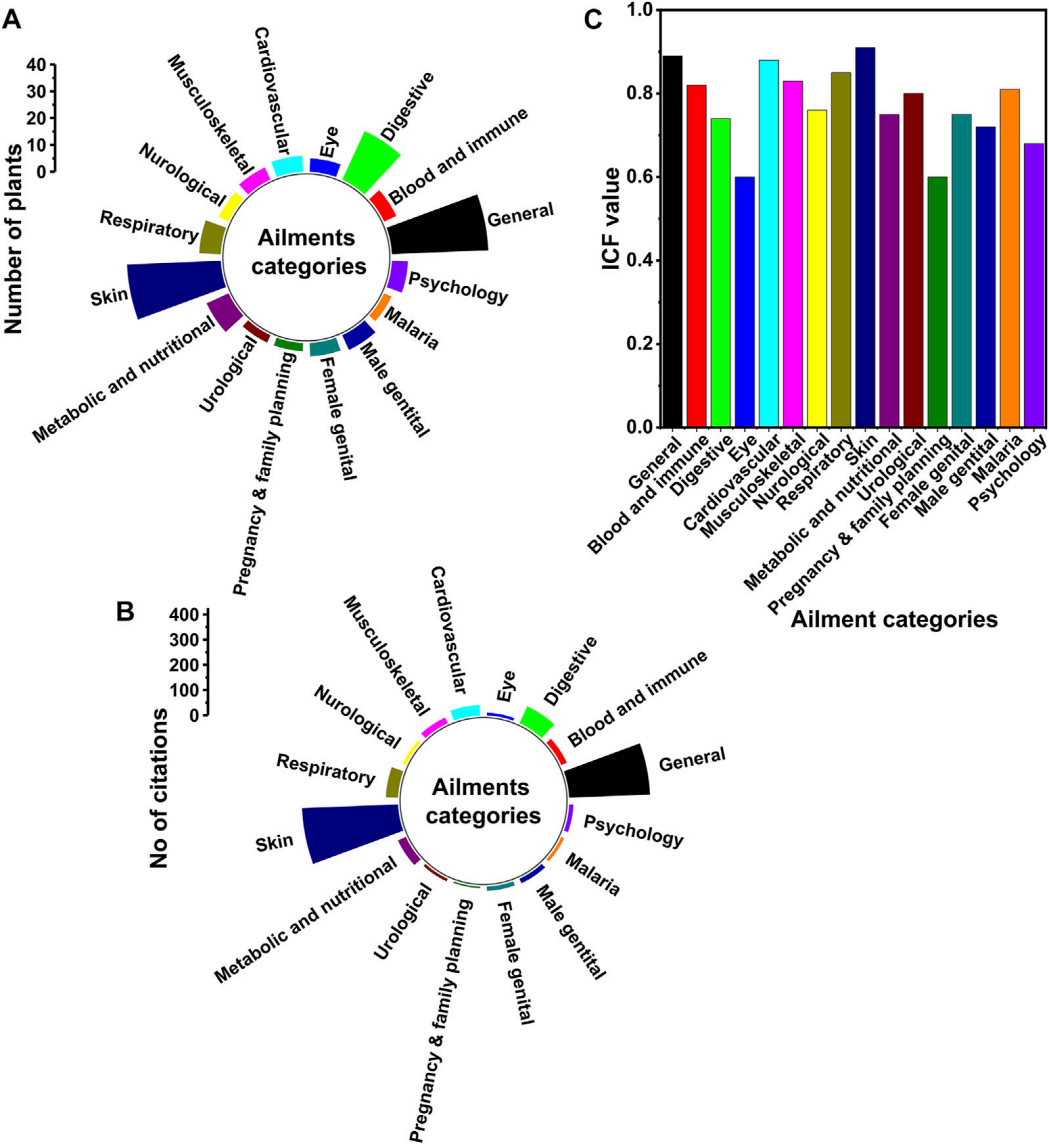
Ailments treated by the traditional healers. The figure depicts the ailments treated by the traditional healers around Gullele botanical garden. **(A)** Ailment categories along with the number of medicinal plants cited for each ailment **(B)** Ailment categories along with the number of citations made by the healers against each ailment **(C)** Informant consensus factor value for each ailment category. The ailments have been categorised as per the International Classification of Primary Care (ICPC-2).

The most common route for administration of the traditional medicine prepared are dermal/topical and oral, having a citation of 43 and 28, respectively (Figure 6B). In addition to dermal, the traditional healers also cited the administration route of the medicine through head rubbing, vaginal and nasal (Figure 6B). In line with our studies, Gidey Yirga also found that dermal is the most common route of administration of the traditional medicine to the patients (Yirga, 2010a). In another study conducted in Hawzen district of northern Ethiopia, the authors found the leading route of administration to be oral, followed by dermal (Yirga et al., 2011). Similarly, in many other ethnobotanical studies conducted anywhere in Ethiopia, the common route of administration is oral (Addis et al., 2001; Kebu et al., 2004). In our study, the predominance of topical or dermal route of administration may be because of the high incidence of skin disease (Figure 7).

### Disease categories and ailments treated by plants

The therapeutic use of the medicinal plants as revealed by the traditional healers has been first recorded during the study in their native language and then translated into English. The ailments treated by the traditional healers have been categorised under the International Classification of Primary Care (ICPC-2) (Staub et al., 2015) (Figure 7). The conversion of the local disease terms to English has been done in the supervision of a medicine practitioner at Addis Ababa. In the current study, most of the medicinal plants have been used to treat several human diseases. This is mainly because of the high prevalence of the human diseases and also due to the dependency of the Ethiopian people on the plant remedies (Dawit, 2001; Bekele, 2007). There are several other studies both in Ethiopia and other countries near the tropics that have shown a high prevalence of medicinal plants used to treat human diseases (Feye and Demissew, 2009; Giday et al., 2009; Abbas et al., 2016). The number of plants used to treat skin and general diseases is almost the same, i.e. 35 and 36, respectively (Table 2 and Figure 7A). Among all the category of diseases, the skin related diseases have the highest citation, i.e. 381 (Table 2 and Figure 7B), followed by the general category, i.e. 321. The general category mainly handles diseases such as headache, wounds, venereal disease, tuberculosis, toothache, plant poison antidote, pain, oedema, internal bruises, inflammation, hives, fever in children, flu-Like Syndrome, fever, faintness, cold, chronic fever, chest operation, cancer, aphrodisiac, antipyretic, antidote, and anthelmintic etc (Table 2 and Figure 7). In addition to this, the other diseases cited by the healers having over 30 citations are digestive (74), respiratory (48), cardiovascular (42) and endocrine diseases (34) (Figure 7B). The number of plants used by the healers to treat these diseases is 20 (digestive), 8 (respiratory), 6 (cardiovascular) and 9 (endocrine) (Figure 7A). Therefore, from the above data, it is quite clear that skin and digestive diseases are more prevalent in the area around Gullele Botanical Garden as compared to other diseases. The number of skin and digestive diseases in the area may be due to the impact of diet and lifestyle of the people around Gullele Botanical Garden. The top three medicinal plants used by traditional healers to treat diseases are *Hagenia abyssinica* (Bruce) J.F.Gmel*.*, *Echinops kebericho* Mesfin and Laggera tomentosa (A.Rich.) Sch.Bip. ex Oliv. & Hiern. The use of these three plants may be because of its abundance and ease of availability in the place. Those plants having high citations against the various diseases should be further explored for their biological activities (Schmeda-Hirschmann et al., 2002). In line with our studies, Giday and co-workers also found that the traditional healers of Bench ethnic group of Ethiopia treated many skin diseases (Giday et al., 2009). The high prevalence of skin diseases around the Gullele Botanical Garden may be because of the prevalence of less sanitation and unhygienic condition.

### Quantitative data analysis

#### Informant consensus factor

The diseases cited by traditional healers have been divided to 16 categories (Figure 7C). The highest informant consensus factor has been recorded for skin diseases, followed by general and cardiovascular diseases (Figure 7C). In addition, the diseases categories having ICF over 0.75 are Blood and immune mechanism, musculoskeletal, neurological, respiratory, endocrine and nutritional, urological, female genital, and malaria (Figure 7C). Digestive and pregnancy, childbearing, and family planning have an ICF value of 0.60, which is the lowest recorded for our study (Figure 7C). Several ethnobotanical studies have been carried out in various places in Ethiopia, having different ICF values for different diseases. In an ethnobotanical study done in northwest Ethiopia, the ICF values for different diseases ranged from 0.04 to 0.84. The ICF value obtained in the study for digestive diseases is like the ICF value obtained in our study (Chekole et al., 2015). In another study done elsewhere in Ethiopia, Tolossa et al. reported the ICF value for various diseases ranged from 1 to 0.72. The ICF values for skin diseases, as reported by Tolossa and co-workers, are in line with our studies (Tolossa et al., 2013). The higher value of ICF mainly indicates that a particular disease is being effectively treated by the local traditional healers. The higher value also shows a substantial degree of agreement between the treatment of diseases using different plant species. The lower value of ICF i.e. less than 0.3 shows that there is less consistency between the knowledge of the traditional healers and the medicinal plants used by them (Teklehaymanot and Giday, 2007). So, for traditional healers, the plants with a variety of medicinal used for multiple diseases are generally considered as being an effective medicinal plant (Bennett and Prance, 2000; Hussain et al., 2019).

### Relative frequency of citation

The relative frequency of citation (RFC) mainly reveals the importance of the medicinal plant species with the reference to the citations provided by the traditional healers (Vitalini et al., 2013). In the current study, the RFC values range from 0.01 to 0.96 (Table 2). It is considered that the medicinal plants having a high RFC value are well known and commonly used by the traditional healers (Kayani et al., 2015). The main reason cited for the high RFC value of medicinal plants is easy availability and a wide distribution. The high RFC medicinal plants should also be explored for bioactive compounds and should be taken by both industry and academia to develop lead drugs and therapeutics for the diseases (Mukherjee and Wahile, 2006). The therapeutic potential of the plants has been recorded since ancient times. The ethnomedicine used by the healers in form of concentrated plant extracts has been used as the guiding polestar by the modern medicine to isolate and purify bioactive compounds against several diseases such as cancer, HIV, diabetes, etc., (Thomford et al., 2018). Traditional medicine as Ayurveda, Unani, Kampo and traditional Chinese medicine have guided us to discover several drugs from the plant extracts and their natural products against many diseases. The anticancer drugs Taxol and Vinblastine from the plants *Taxus brevifolia* and *Catharanthus roseus*, respectively and anti-malarial drugs quinine from *Cinchona* spp. and Artemisinin from *Artemisia annua* are well known drugs isolated from plant extracts for treating the respective diseases (Thomford et al., 2018). The natural products from the plants have been the source of drugs for various diseases and are a great source of antimicrobial agents (Thomford et al., 2018). In course of time the traditional medicine had been subdued by the modern medicine, but in the past few decades the use of medicinal plants in promotion of health and in treating of various diseases has increased (Thomford et al., 2018). In our current study, some of the medicinal plants have bioactivities while the bioactivities of a lot of endemic plants are still to be explored. Therefore, the medicinal plants with high RFC value should be prioritized for conservation and should not be over harvested.

### Fidelity level

The fidelity level (FL) of the medicinal plants used to treat various diseases in this study varies from 50 to 100% (Table 2). The data analysis revealed that three medicinal plants have 100% fidelity. *Echinops kebericho* Mesfin, *Galium abaujense* Borbás and Laggera tomentosa (A.Rich.) Sch.Bip. ex Oliv. & Hiern has 100 percent fidelity and is used to treat snakebite, eczema and leech would heal, respectively (Table 2). Ten medicinal plants exhibited fidelity level over 90 percent. *Cyathula uncinulata* (Schrad.) Schinz, *Jasminum abyssinicum* Hochst. ex DC., *Linum usitatissimum* L., and *Olinia rochetiana* A.Juss. has 90 percent fidelity level in treating wounds (Table 2). Similarly, *Dichrocephala integrifolia* (L.f.) Kuntze and *Phytolacca dodecandra* L’He’r. having 90% FL, is used for the treatment of skin disease i.e. wart. Other plants such as *Carissa spinarum* L., *Clematis simensis* Fresen., *Lactuca inermis* Forssk. and *Sparrmannia ricinocarpa* (Eckl. & Zeyh.) Kuntze is used for the treatment of evil eye, tonsillitis, impotency and hepatitis, respectively (Table 2). Nearly 17 medicinal plants have fidelity level ranging from 80–85 percent (Table. 2). The high level of fidelity for a particular medicinal plant suggests that the medicinal plant is the most preferred plant species to treat a particular disease (Shil et al., 2014). Fidelity level is often treated as a useful ethnobotanical index for the selection of most preferred plants by the traditional healers for the treatment of a disease (Khan et al., 2014). It is generally seen that high FL values medicinal plants have a good reservoir of bioactive compounds and hence are a good target for phytochemical investigations (Hassan-Abdallah et al., 2013). In the current study, over 25 plant species out of 81 have fidelity level of 50 percent (Table 2). The low fidelity level of medicinal plants mainly indicates a less preference of the medicinal plant for treating any ailments (Hussain et al., 2019). The main reason for the low fidelity level is due to the less or limited knowledge of the medicinal plants of the traditional healer for using for the treatment of the diseases(Rehman et al., 2017).

### Comparison of the data with nearby ethnobotanical studies

The similarities and differences in the ethnobotanical studies in a region reveal the indigenous knowledge of the medicinal plants based on the historical, phytochemicals, and ecological factors that govern the selection of medicinal plants (Moerman, 1998; Leonti et al., 2003; Ladio et al., 2007). The current ethnobotanical study has been compared with other ethnobotanical and ethnomedicinal studies in Ethiopia. Jaccard’s Index (JI) analysis of all the seven ethnobotanical and ethnomedicinal studies in Ethiopia with the current study revealed a similarity percentage between 21.43 and 48.19 percent (Table 4). In the current study, the highest similarity percentage of 48.19 and JI of 0.32 is observed for the ethnobotanical study conducted at Debre Libanos district of Ethiopia (Table 4). The high similarity percentage, as well as JI, may be because of the proximity of the study area to Gullele Botanical Garden. Following Debre Libanos district, Wonago district also recorded a high similarity percentage and JI of 43.10% and 0.22, respectively (Table 4). This shows that the plant species and the therapeutic practices adopted by the traditional healers are similar (Shaheen et al., 2017). In addition to this high similarity also reveals a similar type of vegetation and climatic condition in both the places (Esakkimuthu et al., 2018; Faruque et al., 2018). The lowest similarity percentage (21.43%) and JI (0.11) are observed in the ethnobotanical study done by Jima et al. at Berbere district of Ethiopia (Table 4). Low similarity mainly reveals a low degree of social trade between the indigenous people in the past, which has brought differences in the ethnobotanical knowledge in the area (Aziz et al., 2018). In addition to this, geological detachment may also play a role leading to considerable loss and propagation of ethnobotanical information in the areas (Amjad et al., 2017).

**TABLE 4:** Jaccard’s Coefficient of Similarity index with six other areas with respect to plants species composition.

Sample study area	Total medicinal plants	Common medicinal plants	Similarity percentage	Jaccard’s index	References
Berbere District, Bale Zone	70	15	21.43	0.11	Yineger et al. (2008)
Nekemte Town	71	20	28.17	0.15	Suleman and Alemu, (2012)
Debre Libanos District	83	40	48.19	0.32	Aydagnehum and Girma, (2014)
Gimbi District	49	15	30.61	0.13	Abera, (2014)
Wonago District	58	25	43.10	0.22	Mesfin et al. (2009)
Zegie Peninsula	67	23	34.33	0.18	Teklehaymanot and Giday, (2007)
Ankober	135	46	34.07	0.27	Lulekal et al. (2013)

### Pharmacological properties and bioactivities of the Asteraceae medicinal plants used by the traditional healers of Gullele botanical garden

In the current study, the traditional healers have used the maximum number of medicinal plants belonging to the family of Asteraceae. Some of these plants are endemic and are being used extensively by the traditional healers in Ethiopia (Ayalew et al., 2022). Among the 12 Asteraceae medicinal plants used by the healers, six of them have fidelity level ranging from 80 to 100 percent. *Echinops kebericho* Mesfin and *Laggera tomentosa* (A.Rich.) Sch.Bip. ex Oliv. & Hiern has the highest fidelity of 100 percent and is also being explored for many of their pharmacological and bioactivities. In many other ethnobotanical and ethnomedicinal studies carried out in Ethiopia, *Echinops kebericho* Mesfin is used by the traditional healers to treat many other diseases such as black leg, respiratory and liver diseases, cough, headache, scabies, toothache, stomach ache, common cold, sunstroke, tonsillitis, vomiting, gonorrhoea etc (Bitew and Hymete, 2019). In most of the preparation of the ethnomedicinal medicines, the root of the plant is mostly used and in some cases the bulb and the stem are used (Bitew and Hymete, 2019). The safety of *Echinops kebericho* Mesfin has been seen in animal models (Deyno et al., 2020a). Several phytochemical screening studies carried out on *E. kebericho* Mesfin have shown the presence of flavonoids, alkaloids, triterpenoids, saponins and steroids in the in the essential oil extracted from the roots (Abegaz et al., 1991; Toma et al., 2015). In addition, the main compounds present in the essential oil of the root extract is dehydrocostus lactone (Abegaz et al., 1991; Hymete and Afifi, 1997; Tariku et al., 2011). It is seen that the methanolic extract and the essential oil of the tubers exhibits antimicrobial and antimycobacterial activities (Desta, 1993; Ashebir and Ashenafi, 1999; Getachew et al., 2011; Ameya et al., 2016). It also shows significant activities against *M. smegmatis* and the fungi like *Aspergillus flavus* and *Candida albicans* (Ameya et al., 2016). The extract obtained from this medicinal plant have also shown activities against malarial parasite, Leishmania, earthworm, and Trypanosoma (Hymete and Kidane, 1991; Tariku et al., 2011; Toma et al., 2015; Ameya et al., 2016). The essential oil obtained from this plant exhibited mosquito repellent and larvicidal activity (Debela et al., 2007; Jemberie et al., 2016). Deyno et al. have shown that the tuber extract of this medicinal plant in five different solvents showed variable MIC concentration against eight bacterial species (Deyno et al., 2020b). In the same study they have also shown the antibacterial activity of essential oil of extracted from *E. kebericho* Mesfin against eight bacteria. Next to *E. kebericho* Mesfin, *Laggera tomentosa* (A.Rich.) Sch.Bip. ex Oliv. & Hiern also has a fidelity of 100 percent. This medicinal plant is endemic to Ethiopia and is used by many traditional healers in Ethiopia. The plant’s crushed juice is taken for the treatment of migraine while the vapor of the boiled leaves is taken to treat cold (Getahun et al., 2019). The aerial part of this plant can be used to treat to many diseases such as toothache, swelling, ringworm, as fumigant, to treat cough and flu (Getahun et al., 2019). It is seen that the polar and the non-polar extract of the aerial part of this plant contains sesquiterpenes and flavones (Hunde and Asfaw, 2015; Getahun et al., 2019). The essential oil isolated from this endemic plant has a different composition than the other species of this genus (Asfaw et al., 2003). The major compounds isolated from the essential oil of *L. tomentosa* i.e. filifolone, isochrysanthenone and chrysanthenone. The aerial methanolic extract from L. tomentosa also exhibits antimicrobial activity against the strains *Neisseria gonorrhoea*, *Streptococcus pyogenous* and *Stretococcus pneumonia* (Geyid et al., 2005). *Dichrocephala integrifolia* (L.f.) Kuntze and *Lactuca inermis* Forssk. have a fidelity of 90 percent. *Dichrocephala integrifolia* (L.f.) Kuntze in addition to Ethiopia is used all over Africa as a traditional medicinal plant to treat schizophrenia, diarrhoea, amebiasis, epilepsy, worm infections, dementia, asthma, malaria, inflammation, hepatitis and gastrointestinal diseases (Enogieru and Momodu, 2021). Several pharmacological and bioactivities have been reported for this medicinal plant. Mothana et al. have shown that *Dichrocephala integrifolia* exhibited antiplasmodial, antileishmanial, and antitrypanosomal activity against *P. falciparum*, *L. infantum*, *T. cruzi*, and *T. brucei* (Mothana et al., 2014). Fankem et al. have shown that the dichloromethane fraction of *D. integrifolia* protects the tissue damage from *S. typhi* infection by preventing the oxidative reactions (Fankem et al., 2019). It is seen that decoction made from the leaves of the plant is able to prevent memory impairment and improves the learning capacities in mice (Enogieru and Momodu, 2021). *Lactuca inermis* Forssk. is a species which is mainly found in tropical and South Africa. A detailed study on the chemical constituents found in this plant and have shown that the coumarins especially scopolin are found in the roots and aerial parts of this plant (Abdel Bar et al., 2022). Although a lot of pharmacological work has been done, *Lactuca sativa* sparse works are done on *L. inermis* (Abdel Bar et al., 2022). *Gymnanthemum amygdalinum* (Delile) Sch.Bip. a native of Africa has been extensively used in traditional medicines for treating several diseases such as constipation, wounds, scabies, tonsillitis, fever, worms infection etc (Kaur et al., 2019). *G. amygdalinum* extracts have shown anti-cancer activity against the MCF-7 and MDA-MB-231 cancer cell lines (Wong et al., 2013). The flavonoids rich fraction of the leaf extracts has shown significant anti-diabetic activity and the possible mechanism of this activity may be due to the regeneration of the pancreatic beta cells (Ebong et al., 2006). The ethanol extract of this plant exhibited anti-malarial activity against the parasite *P. berghei* and the aqueous extract against *P. falciparum*, *P. vivax* and *P. ovale* (Odeh and Usman, 2014; Omoregie and Pal, 2016). In the animal models it is seen that the extracts of the plants reduces the inflammation significantly (Adedapo et al., 2014). In addition to this the plant extracts provides significant hepatoprotective and anti-pyretic activity (Kaur et al., 2019). The extract of the whole plant of *G. amygdalinum* shows anti-bacterial activity against the bacteria *S. aureus*, *P. aeruginosa*, and *E. coli* (Adetunji et al., 2013). In the same study the authors have shown that the hot extract from the plant exhibited the highest zone of inhibition against the bacterium *P. aeruginosa*. In another study it is shown that the ethanolic extract of the plants exhibits greater bactericidal activity against *S. mutans* (Anibijuwon et al., 2012). In other studies, the aqueous and the methanolic extract of the plant shows significant analgesic and hypolipidaemic activity (Anibijuwon et al., 2012). In addition, this the extracts form the plant also exhibits anti-oxidant, sedative and anti-leishmanial activity (Kaur et al., 2019). *Inula acaulis* Schott & Kotschy ex Boiss. has a fidelity of 80 percent. Although a lot of pharmacological activities are carried out on the genus Inula but very few works are carried out on this particular medicinal plant. Hence there lies a scope to explore the bioactivity and pharmacological properties of this plant. Similarly, *Erigeron steudelii* (Sch.Bip. ex A.Rich.) Sch.Bip. ex Schweinf. is also less explored which is used for treating skin wounds and has a fidelity of 75 percent. *Tagetes minuta* L. has a fidelity of 70 percent and is used to treat a number of diseases. It is also used in food and aromatherapy because of the unique composition of its essential oil (Gakuubi et al., 2016). The essential oil of *T. minuta* shows antibacterial activity against a range of human, plant and animal pathogenic bacteria (Gakuubi et al., 2016). It is seen that the major component of the essential oil exhibiting the antibacterial activity are (Z)-βocimene and dihydrotagetone (Smith et al., 1998; Trombetta et al., 2005). In the same studies the authors have also shown that the essential oil is more sensitive towards Gram-positive bacteria than towards the Gram-negative bacteria. The essential oil from *T. minuta* have exhibited considerable antifungal activity against a wide range of fungi (Matasyoh et al., 2007; Marei et al., 2012). The essential oil of this plant also exhibits insecticidal activity against head lice and human ectoparasite (Cestari et al., 2004). In addition to this the essential oil is also active against a variety of other insects (Gakuubi et al., 2016). It also exhibits acaricidal and repellent activity against ticks and mosquitos (Gakuubi et al., 2016). *T. minuta* essential oil also shows nematocidal activity against many plant parasites (Gakuubi et al., 2016). In addition to these all bioactivities it also shows excellent antioxidant and anti-cancer activities. The components of essential oil which are supposed to exhibit strong anti-oxidant activity are (*E*)-β-ocimene, L-verbenone and limonene (Gakuubi et al., 2016). Similarly, the essential oil of this plant exhibits anti-cancer activity against a variety of cell lines such as NB4, EACC, HL-60, HepG2 etc (Gakuubi et al., 2016). *Solanecio gigas* (Vatke) C.Jeffrey is an endemic plant of Ethiopia and has been used to treat many diseases such as diarrhoea, wounds, skin and liver diseases (Ayalew et al., 2022). As this plant is endemic to Ethiopia a very few studies have been carried out to characterize the pharmacological and bioactivities of this plant. The dichloromethane and methanol extract of the flower of this plant exhibits antitrypanosomal activity against *T. brucei* and cytotoxic activity against HL cell lines (Nibret et al., 2009). In another study it is shown that the dichloromethane and the acetone extract of the roots of *S. gigas* showed antiviral activity against HIV-1 and HIV-2 (Asres et al., 2001). *Bidens pilosa* L. is a medicinal herb which is mostly used in tea and medicines. This plant is versatile and different parts of this plant are used to treat several numbers of ailments all over the world (Bartolome et al., 2013a). Many research works have demonstrated that the extracts of *B. pilosa* shows various bioactivities. The aqueous and alcoholic crude extracts of this plant exhibit anti-tumor activity in animal models (Kviecinski et al., 2008). Kumari and co-workers also showed that the leaves extract of this medicinal plants exhibited anti-cancer activity (Kviecinski et al., 2008). The extract from the dried powder of the aerial part of this plant have shown anti-inflammatory effect in mice models (Horiuchi and Seyama, 2008). In the same study the authors have proposed that the anti-inflammatory effect may be due to the phenolics in the plant extract. Some of the major phenolics present in the plant are luteolin and ethyl caffeate (Bartolome et al., 2013a). *B. pilosa* has been used as an anti-diabetic herb in various parts of the world (Bartolome et al., 2013a). It is seen that anti-diabetic activity in mainly due to the polyynes present in the plant (Chang et al., 2007). In addition to these bioactivities, the essential oil from the flowers and the plant extracts of *B. pilosa* exhibits significant antioxidant activity (Deba et al., 2008). In the same study the authors have shown that combined antioxidant effect of the essential oil from the leaves and flowers is more than the antioxidant activity of the flowers only. The essential oil and the extract from the leaves and flowers of this plant exhibited antibacterial activity against six bacteria (Deba et al., 2008). The methanolic and acetone extracts of the roots from the plants also exhibited anti-bacterial activity (Ashafa and Afolayan, 2009). In addition to the anti-bacterial activity, the hot water extracts of the roots, stems and leaves showed anti-fungal activity against the fungi *Corticium rolfsii*, *Fusarium solani,* and *Fusarium oxysporum* (Deba et al., 2007). The plant extracts are also known to possess vasodilatory and wound healing activities (Bartolome et al., 2013b). *Orbivestus leopoldii* (Sch.Bip. ex Walp.) H.Rob. is plant which is mainly used in the middle east countries to treat diseases like cough and skin. The pharmacological and bioactivities of this plant has not been explored extensively. It is seen that the extract from this plant exhibits antiprotozoal, cytotoxic and antimicrobial activities (Toyang and Verpoorte, 2013). *Pentanema confertiflorum* (A.Rich.) D.Gut.Larr., Santos-Vicente, Anderb., E.Rico & M.M.Mart.Ort has the least fidelity of 50 percent among all the Asteraceae plants. *Pentanema confertiflorum* is used in various regions of Ethiopia for traditional medicine (Ayalew et al., 2022). The methanolic extracts of the leaf and flower of this plant exhibits antimicrobial activity against *S. aureus* and *T. mentagrophytes* (Messele et al., 2004). In another study the leaf extracts from this plant shows antiviral and cytotoxic activities against the virus HSV-1, Influenza A and Coxsackievirus B3 & cancer cell lines HeLa, MDCK and GMK, respectively (Gebre-Mariam et al., 2006). Therefore, from the above discussion it is clear that the pharmacological and bioactivities of most of the Asteraceae family medicinal plants used by the traditional healers of Gullele Botanical Garden has been explored. But still there are a few endemic plants which are less explored. In addition to this there are many bioactivities and numerous other applications of these plant extracts such as in green synthesis, elucidation of bioactive structure, etc which are still to be explored.

## Conclusion

To the best of knowledge, this is the first systematic qualitative and quantitative ethnobotanical and endemism study conducted in Gullele Botanical Garden in Addis Ababa, Ethiopia. The medicinal plant species documented in the current study are used to treat a variety of human ailments. Most of the medicinal plants are herbs and shrubs and for preparing the traditional medicine by the healers. Mostly the leaves of the medicinal plants are used. Most herbal preparation is made from fresh medicinal plants, majorly by crushing and pounding. This study also reveals that the most treated ailments by the healers are skin and, as expected, the most cited route of administration of the medicine is dermal/topical. This study also reveals that there are several endemic plants in the garden, which are still unexplored by the traditional healers. The medicinal plants used by the traditional healers around Gullele Botanical Garden to treat the patients in the area through traditional healthcare systems significantly complement the modern healthcare systems, as the later are considerably costlier. The use of these medicinal plants by the healers lowers the cost of the traditional medicines, which can be easily accessed by the ethnic groups around the garden. In addition, the use of the plant-based medicines by the healers helps in the continuation of the practice of the traditional medicinal knowledge as well as transfers this information of the traditional medicine to the young generation. There are various medicinal plants with high citations for which further phytochemical, pharmacological, microbiological, and toxicological investigations are needed. In addition, this study reports many endemic plants in GBG having medicinal value but are either endangered or vulnerable. To conserve these endemic medicinal plants the GBG authorities should propagate and conserve these endemic plant species. Besides this, due to increasing modernization, the traditional medicinal knowledge is eroding at a very fast pace and will be extinct soon. Hence, endless, and tireless efforts are needed to conduct more ethnobotanical and ethnomedicinal studies both in Ethiopia and throughout the world to document and preserve the precious and invaluable traditional medicinal knowledge.

## Data Availability

The original contributions presented in the study are included in the article/Supplementary Material, further inquiries can be directed to the corresponding authors.
