# Supplementary material for: Ethnobotanical study of endemic and non-endemic medicinal plants used by indigenous people in environs of Gullele botanical garden Addis Ababa, central Ethiopia: A major focus on Asteraceae family
Source: Front Pharmacol. 2022 Nov 23;13:1020097. doi: 10.3389/fphar.2022.1020097 (PMC9727095; doi:10.3389/fphar.2022.1020097)
Supplement: Supplementary file 1 [file DataSheet1.docx]

Ethnobotanical study of endemic and non-endemic medicinal plants used by indigenous people in environs of Gullele Botanical Garden Addis Ababa, central Ethiopia

Melaku Masresha Woldeamanel^1,2^, Mohammed Kasso Geda^2^, Shibani Mohapatra^1^, Tapan Kumar Bastia^1^, Prasanta Rath^1*^ and Alok Kumar Panda^1*^,

^1^School of Applied Sciences, Kalinga Institute of Industrial Technology, Deemed to be University, Bhubaneswar-751024, India

^2^Department of Biology, College of Natural and Computational Sciences, Dire Dawa University, Dire – 138, Ethiopia

*** Correspondence:**Corresponding Authors
[alok.pandafch@kiit.ac.in](mailto:alok.pandafch@kiit.ac.in) (Alok Kumar Panda) and [prathfch@kiit.ac.in](mailto:prathfch@kiit.ac.in) (Prasanta Rath)

Keywords: Ethiopia, Ethnobotany, Endemism, Indigenous botanical knowledge, Medicinal plants, Gullele Botanical Garden, Addis Ababa

Table S1. Detailed sociodemographic information of the traditional healers.

| **No of healers** | **Sex** | **Age** | **Marital Status** | **Educatio n** | **Residence** | **Occupation** | **Ethics group** | **Religion** | **Numbe r**  **of plants** | **Detailed**  **informant information** |
| --- | --- | --- | --- | --- | --- | --- | --- | --- | --- | --- |
| 1 | F | 24 | Unmarried | Modern | Mullo | Merchant | Oromo | Orthodox | 26 | TN0-1 |
| 2 | M | 33 | Married | Modern | Sululta | Merchant | Oromo | Orthodox | 26 | TN0-2 |
| 3 | M | 44 | Married | Modern | Sabata | Merchant | Amhara | Protestant | 31 | TN0-3 |
| 4 | F | 46 | Married | Religious | Sululta | Farmers | Amhara | Orthodox | 29 | TN0-4 |
| 5 | M | 56 | Divorce | Uneducated | Sululta | Farmer | Tigray | Orthodox | 28 | TN0-5 |
| 6 | M | 23 | Unmarried | Modern | Sabata | Student | Amhara | Orthodox | 30 | TN0-6 |
| 7 | M | 30 | Married | Uneducated | Mullo | Farmer | Amhara | Orthodox | 32 | TN0-7 |
| 8 | F | 43 | Divorce | Religious | Sebeta | Farmer | Oromo | Muslim | 29 | TN0-8 |
| 9 | M | 54 | Married | Religious | Sebeta | Healer | Oromo | Orthodox | 37 | TN0-9 |
| 10 | M | 42 | Married | Modern | Sebeta | Merchant | Amhara | Orthodox | 30 | TN0-10 |
| 11 | M | 34 | Married | Modern | Sululta | Merchant | Oromo | Protestant | 29 | TN0-11 |

| 12 | M | 21 | Unmarried | Modern | Welmera | Student | Gurage | Muslim | 29 | TN0-12 |
| --- | --- | --- | --- | --- | --- | --- | --- | --- | --- | --- |
| 13 | F | 37 | Married | Modern | Lagatafo | Healer | Amhara | Orthodox | 29 | TN0-13 |
| 14 | M | 47 | Married | Modern | Lagatafo | Merchant | Amhara | Protestant | 27 | TN0-14 |
| 15 | M | 33 | Unmaried | Modern | Walmara | Merchant | Amhara | Orthodox | 30 | TN0-15 |
| 16 | M | 22 | Unmarried | Modern | Sululta | Merchant | Oromo | Muslim | 31 | TN0-16 |
| 17 | F | 55 | Divorce | Uneducated | Lagatafo | Healer | Oromo | Muslim | 33 | TN0-17 |
| 18 | M | 60 | Married | Religious | Sululta | Farmer | Oromo | Muslim | 30 | TN0-18 |
| 19 | M | 32 | Unmarried | Modern | Sululta | Merchant | Oromo | Muslim | 31 | TN0-19 |
| 20 | F | 20 | Unmarried | Modern | Burayu | Students | Amhara | Orthodox | 30 | TN0-20 |
| 21 | M | 41 | Married | Modern | Sabata | Farmer | Gurage | Muslim | 32 | TN0-21 |
| 22 | F | 53 | Married | Religious | Sululta | Farmer | Sidama | Protestant | 35 | TN0-22 |
| 23 | M | 61 | Married | Uneducated | Mullo | Farmer | Gurage | Muslim | 34 | TN0-23 |
| 24 | M | 58 | Married | Uneducated | Mullo | Farmer | Amhara | Orthodox | 35 | TN0-24 |
| 25 | M | 25 | Unmarried | Modern | Sululta | Farmer | Amhara | Orthodox | 32 | TN0-25 |
| 26 | F | 48 | Married | Modern | Sululta | Farmer | Sidama | Protestant | 29 | TN0-26 |
| 27 | M | 57 | Married | Religious | Lagatafo | Farmer | Amhara | Orthodox | 29 | TN0-27 |
| 28 | M | 31 | Unmarried | Modern | Walmera | Farmer | Dawro | Protestant | 32 | TN0-28 |
| 29 | F | 62 | Married | Modern | Sululta | Farmer | Oromo | Orthodox | 32 | TN0-29 |
| 30 | M | 91 | Married | Religious | Sululta | Healer | Oromo | Muslim | 38 | TN0-30 |

| 31 | M | 37 | Married | Modern | Sabata | Farmer | Oromo | Orthodox | 27 | TN0-31 |
| --- | --- | --- | --- | --- | --- | --- | --- | --- | --- | --- |
| 32 | F | 74 | Married | Modern | Sabata | Healer | Amhara | Orthodox | 32 | TN0-32 |
| 33 | M | 66 | Married | Religious | Lagatafo | Farmer | Oromo | Orthodox | 32 | TN0-33 |
| 34 | M | 72 | Married | Uneducated | Mullo | Healer | Oromo | Orthodox | 35 | TN0-34 |
| 35 | M | 24 | Unmarried | Modern | Mullo | Student | Oromo | Protestant | 24 | TN0-35 |
| 36 | F | 52 | Divorce | Modern | Sabata | Farmer | Oromo | Protestant | 28 | TN0-36 |
| 37 | M | 61 | Married | Uneducated | Lagatafo | Farmer | Oromo | Muslim | 28 | TN0-37 |
| 38 | M | 63 | Married | Uneducated | Akaki | Farmer | Tigri | Orthodox | 28 | TN0-38 |
| 39 | F | 30 | Unmarried | Modern | Sabata | Merchant | Amhara | Orthodox | 27 | TN0-39 |
| 40 | M | 49 | Married | Modern | Sabata | Merchant | Amhara | Orthodox | 27 | TN0-40 |
| 41 | M | 58 | Married | Uneducated | Sululta | Farmer | Amhara | Orthodox | 23 | TN0-41 |
| 42 | M | 64 | Married | Uneducated | Mullo | Farmer | Amhara | Orthodox | 21 | TN0-42 |
| 43 | F | 38 | Unmarried | Modern | Sabata | Merchant | Gurage | Muslim | 23 | TN0-43 |
| 44 | M | 60 | Married | Uneducated | Sululta | Farmer | Sidama | Protestant | 23 | TN0-44 |
| 45 | M | 81 | Married | Religious | Lagatafo | Healer | Oromo | Muslim | 20 | TN0-45 |
| 46 | F | 29 | Married | Modern | Sululta | Merchant | Amhara | Orthodox | 22 | TN0-46 |
| 47 | M | 50 | Married | Modern | Sabata | Farmer | Tigri | Orthodox | 21 | TN0-47 |
| 48 | F | 68 | Married | Religious | Sabata | Healer | Oromo | Orthodox | 21 | TN0-48 |
| 49 | M | 79 | Married | Uneducated | Sululta | Farmer | Amhara | Orthodox | 20 | TNO-49 |
| 50 | M | 26 | Unmarried | Modern | Lagatafo | Merchant | Amhara | Orthodox | 19 | TN0-50 |
| 51 | F | 59 | Married | Modern | Mullo | Healer | Gurage | Muslim | 16 | TN0-51 |
| 52 | M | 69 | Married | Uneducated | Walmara | Farmer | Amhara | Orthodox | 21 | TN0-52 |
| 53 | M | 71 | Married | Uneducated | Sabata | Healer | Amhara | Orthodox | 27 | TN0-53 |
| 54 | F | 27 | Unmarried | Modern | Lagatafo | Student | Oromo | Orthodox | 16 | TN0-54 |
| 55 | M | 39 | Married | Modern | Mullo | Farmer | Oromo | Orthodox | 14 | TN0-55 |
| 56 | F | 59 | Married | Modern | Sululta | Farmer | Oromo | Muslim | 17 | TN0-56 |
| 57 | M | 62 | Married | Modern | Sabata | Farmer | Amhara | Orthodox | 16 | TN0-57 |
| 58 | M | 83 | Married | Uneducated | Sululta | Healer | Amhara | Orthodox | 23 | TN0-58 |
| 59 | F | 40 | Married | Modern | Mullo | Farmer | Walita | Protestant | 15 | TN0-59 |
| 60 | M | 28 | Unmarried | Modern | Lagatafo | Farmer | Amhara | Orthodox | 19 | TN0-60 |

Table S2. Detail of the methods adopted by the traditional healers for the preparation of the medicines.

| **Local Name** | **Botanical Name** | **Family** | **PU** | **RA** | **Disease Indication** | **Methods adopted by traditional healers for the preparation of the medicine** |
| --- | --- | --- | --- | --- | --- | --- |
| *Sessa* | *Alchemilla pedata* Hochst.ex A.Rich | Rosaceae | Wh | Dr | Common cold, Thyroid, anemia, depression or anxiety | The plant is crushed, then dissolved in a little quantity of water and filtered. To cure the common cold, typhoid fever, and anemia, one must drink one cup of the solution on an empty stomach, or one cup of the solution mixed with one cup of the local alcohol "Hereqe," or one cup of coffee every day for one week. One must smoke the whole plant to treat sadness and anxiety. |
| Eret | *Aloe percrassa* Tod. | Asphodelaceae | L | Dr,O,E | Hemorrhoids, remove dead skin from hair (Dandruff), malaria, eye disease, wound, hair lose | One tablespoon of the dried leaves should be added to one liter of boiling water, and then one glass of the resulting infusion should be consumed three times a day for one week. Additionally, fresh leaves could be crushed and used topically in order to treat hemorrhoids and dandruff.  In addition, consume one cup of the juice in order to treat malaria, and use the leaves to clean the area around the spiked eye. |
| *Sariitii* | *Asparagus botswanicus* Sebsebe | Asparagaceae | R, L | O | STDs (L), impotence(L), hemorrhoids (R) | A mixture of crushed leaves and water is homogenated and 1 glass consumed for prevention of STD and decrease in impotence. The paste of the roots is inserted anally for hemorrhoid. |
| Azamir | *Bersama abyssinica* Fresen. | Francoaceae | L |  | Diabetes mellitus | Add to water the crushed leaf and make 1 cup of decoction to drink for five days. |
| *Yesetan merfe* | *Bidens pilosa* L. | Asteraceae | L | O | Malaria & fungal | Crushed, squeezed, dripped into skin/ear for removal of fungal infection.and The lead is powdered before being mixed with water and then drink one cup every five days for five days for malaria. |
| *Sanaafica* | *Rhamphospermum nigrum* (L.) Al-Shehbaz | Brassicaceae | Se | o | Gout arthritis | Taken with the seeds of Nigella sativa L. chewed & swallowed |
| *Qumanya,Tibira* | *Brucea antidysenterica* J.F.Mill. | Simaroubaceae | L | O | Diarrhoea | Chewed along with a leaf of *Myrsine africana* L. and swallowed |
| *Adaaddo/anfaari* | Buddleja polystachya Fresen. | Scrophulariaceae | R,L | Ha, Dr | Evil eye | Crushed and squeezed onto the palms, the concentrate is sucked through the nose to relieve severe headaches caused by evil eyes. |
| *Agam* | *Carissa spinarum* L. | Apocynaceae | Rb | N | Evil eye | The root bark is extracted with 1 cup of water and is drunk for 2 days with crushed leaves of *Olinia rochetiana* A.Juss., *Osyris quadripartita* Salzm. ex Decne., and *Juniperus procera* Hochst. ex Endl.. |
| Zikita | Calpurnia aurea (Aiton) Benth. | Fabaceae | L,R | Dr | Ascariasis (L),  rabies (R),  gland cancer (R) | The crushed leaves are extracted with water, one-half cup is taken for Ascariasis. Root are crushed up and dissolved in water, and one cup is consumed three times daily for seven days for Rabies. A longer segment of the root is placed on a square of fabric, and the resulting amulet is worn around the neck for gland cancer. |
| *Gura antuta* | *Centella asiatica* (L.) Urb. | Apiaceae | L | O, N | Headache,  damaged Skin, lose color (vitiligo) | Three drops of leaf juice are dripped into the inner ear to treat headaches, and a crushed leaf mixed with butter is applied to the spots of damaged skin color. |
| *Yazo egier* | *Clematis simensis* Fresen. | Ranunculaceae | L | D | Tonsillitis, rheumatic,  wounds, bloats,  engraving skin with iron | Crushed and concentrated solution poultice applied on site; also, a weekly dose of one cup of leaf water extract is consumed for wound and Rheumatic. Drink 1 cup for leaf juice for Tonsillitis |
| *Misrich* | *Clerodendrum* *abilioi* R.Fern. | Lamiaceae | R,L | N, Dr | Gonorrhea (R), rabies(L), measles(L), tuberculosis (R), colic (L), eye disease,(L) malaria(L), swellings in the body (L), wound dressings(L), asthma (R) and anaphrodisiac (R),  inducing fertility | For gonorrhea, an infusion of one glass of leaves, taken five times in the morning, Crushed parts of the root and leaf are mixed in water, and one 'kubaya' or glass of the mixture is consumed once a week for a period of three weeks in order to stimulate fertility. For relief from abdominal discomfort and Asthma, the root is ground into powder, then diluted in salty water, and one kubaya is taken. Crushed, moist paste is applied to the affected section of wounds and is used topically. |
| *Anccote* | *Clutia abyssinica* Jaub.& Spach | *Peraceae* | Wh | Dr | Wound | Crushed seeds are applied on wound or chewed. |
| *Yehiya anjet* | *Commelina benghalensis* L*.* | Commelinaceae | St | Dr | Infertility, burns, sore throats, sore eyes, diarrhoea with blood and mucus (dysentery), rashes, and leprosy, ring worm | Pieces of stem and leaves are crushed and dissolved in water; for infertility, one "kubaya" or glass is consumed once a week for a period of three weeks. After being wrapped in the leaf and roasted over fire, the wet concentrate solution is smeared or painted over the area of the body that has leprosy, burns, or rashes. Crushed stem dissolved in water, and half a bottle of it is consumed twice a week to treat sore throats. |
|  | *Erigeron steudelii (Sch.Bip. ex A.Rich.) Sch.Bip. ex Schweinf.* | Asteraceae | L | Dr | Wound | Leaves are crushed between the handsand a viscous extract poured over the wounds. |
| *Chegogot* | *Cyathula uncinulata*  (Schrad.) Schinz | Amaranthaceae | L | Dr | Wound | On the spot, the leaf are shattered, and paste are prepapred and spread. |
| Astenagirt | *Datura stramonium* L. | Solanaceae | L | O,Dr | Stomach, intestinal pain, toothache, and fever, scalp, to treat dandruff and falling hair. | Chewing a couple of leaves and holding the liquid in mouth help alleviate dental pain. Crushed leaf and water extract of one cup is consumed for stomach and intestinal discomfort; washing hair with crushed leaf once a week helps cure dandruff and other hair problems. |
|  | *Delphinium wellbyi* Hemsl. | Ranunculaceae | R,Fl | Dr, O | Common cold (Fl) ,anaemia (R) | To treat a cold and anemia quickly and effectively, one cup of flower and root juice is consumed first thing in the morning. |
| *Sonshi* | *Dichrocephala integrifolia* (L.f.) Kuntze | Asteraceae | L | Dr | Wart | The paste of the crushed plant leaves is applied on dead skin |
| *Alimt* | *Discopodium penninervium* Hochst. | Solanaceae | L | Hd | Prolonged labor | One cup of the leaf extract is consumed until the condition improves. |
| Kitkita | *Dodonaea viscosa* subsp. *angustifolia* (L.f.) J.G.West | Sapindaceae | L | O | Stomach, intestinal pain, toothache, and fever, scalp, dandruff, and falling hair. | Concoction that has been crushed and mixed with the leaf and root bark of *Leucas calostachys* Oliv. and *Brucea antidysenterica* J.F.Mill. is used for intestinal pain. Powdered was kept on toothache for five minutes. One can was consumed for dhariea because of stomach and intestinal discomfort. Powdered was combined with water to form a paste that was applied on scabs, dandruff, and hair loss. |
| *Koshommi/anqaqute* | *Dovyalis abyssinica* (A.Rich.) Warb. | Salicaceae | R,L | O,Dr | Indigestion (R,L) and,increase life expectancy (R,L) |  |
| *Kebericho* | *Echinops kebericho* Mesfin | Asteraceae | R | D | Migraine,mental illness, heart pain, lung TB, leprosy, diarrhoea, kidney, malaria, bilharzias, and amoebic, fever, stomach-ache, and cough, mosquitoes and as a snake repellent, inhaled to fight typhus and fever. | Root pieces are grounded up, a decoction is made, buttered, and one cup is consumed each morning for the next five days to treat a cough. Inhalation of root smoke for the treatment of migraines, mental disease, and smoke also inhaled for fever as well as for evil spirit. To avoid snake smoke is given in house.For sudden abdominal pain root is chewed along with salt and swallowed. The root smoke is inhaled by covering the smoke to avoid evil spirit. |
| *Sombbo/Duduna* | *Ekebergia capensis* Sparrm. | Meliaceae | Sb | O | Lung, tuberculosis syphilis and seizure | To treat lung tuberculosis, stem bark latex is collected, boiled together with bread of *Ensete ventricosum* (Welw.) Cheesman , and eaten. The root of the plant is also wrapped in a piece of clean fabric as an amulet and worn around the neck. This treatment is continued for five days continuously. 1 cup of the aqueous extract of the stem bark is taken in order to cure syphilis and seizures |
| *Haanquu* | *Embelia schimperi* Vatke | Primulaceae | L | Dr, O | Infertility, trachoma | The leaves of the plant is crushed and combined with the dried fruits of the *Hagenia abyssinica* (Bruce) J.F.Gmel. plant, then diluted in water and left to ferment overnight; the following day, one tin of the resulting concoction is consumed on an empty stomach to treat infertility, and it can be applied to the eyes to treat trachoma. |
| *Barzaafi adi* | *Eucalyptus globulus* Labill. | Myrtaceae | Fi, Fr | O | Chronic diarrhoea , headache | Internal fiber and fruits are boiled together and a small amount in the form of juice is taken for diarrhoea and steam of boil for headache. |
| *Qulqualda* | *Eulophia streptopetala* Lindl. | Orchidaceae | Wh | O | General weakness (supply energy especially for people living with HIV virus) | To get strength the whole plant is used in the form of meal as well as in the form of juice during morning time. |
| *Ashkita* | *Galium abaujense* Borbás | Rubiaceae | St,L | Dr | Eczema (chife) (St or L) | Stem or leaf of the plant powder is pasted on itchy skin and rashes for hours |
| *Heexo* | *Hagenia abyssinica*  (Bruce) J.F.Gmel. | Rosaceae | Fb | O | Tapeworm, fracture making | Flower bunches are mingled in. Animal corm is cut and fed till it improves fracture and a solution of boiled flower bunds is consumed to remove tapeworm; human corm is cooked and eaten together with yoghurt "irgo" or a local fermented beverage called "borde".. |
| *Hin’dHe,Garam’bba* | *Hypericum revolutum* Vahl | Hypericaceae | L,Se | O, N | Evil eye, Jaundice, & Headache | Crushed seeds are homogenized with a little amount of water and consumed for jaundice. A solution of the leaves is poured over the head to treat headaches and evil eye. |
| *Arem* | *Hypoestes forskaolii*  (Vahl) R.Br. | Acanthaceae | L | Dr | Dandruff | Crushed and wet concentrate solution is painted on the site of the dandruff with oil for one week |
| *Inshoshilla* | *Impatiens rothii* Hook.f. | Balsaminaceae | L | HD | Dandruff | Powder and wet concentrate solution is painted on the site of the dandruff with butter for three days. |
| Woynagift | *Pentanema confertiflorum* (A.Rich.) D.Gut.Larr., Santos-Vicente, Anderb., E.Rico & M.M.Mart.Ort. | Asteraceae | R | Dr | Fungus | *Nicotiana tabacum* L. is pulverized along with the roots, and the solution is inhaled via the nasal passages. The roots are crushed and combined with the leaf of *Premna schimperi* Engl. and the root of *Clerodendrum myricoides* (Hochst.) R.Br. ex Vatke in order to eradicate fungus.The combination should be soaked in water. |
|  | *Inula acaulis* Schott&Kotschy ex Boiss | Asteraceae | L | Dr | Epistaxis | Leaf crushed, homogenized in little water  and filtered and inhaled and drunk during nose bleeding. |
| *Habte harege/*   \| Tenbelel \| \| --- \| | *Jasminum abyssinicum*  Hochst. ex DC. | Oleaceae | L | Dr | Wound | Leaf pulverised and mixed in enough water together and the solution is spread over the infected skin |
| *Gattiraa ,hindhessa* | *Juniperus procera*  Hochst. ex Endl. | Cupressaceae | R,Sb,Fr | O | Haematuria (blood in the urine (R,Sb&Fr) | Root, stem, and bark powder, together with flower powder is added to the decoction made from *Cupressus lusitanica* Mill. leaf and 1 glass is consumed each day, either by itself or mixed with oatmeal and consumed. |
| *Endawula* | *Kalanchoe petitiana* A.Rich*.* | Crassulaceae | L,Wh.R | O | Swelling by heating(L), gonorrhoea (Wh), syphilis(R) | Whole plant, as well as leaf crushed and extracted with water is used to treat gonorrhea. A few pieces of the root are eaten, and the fluid is retained in the mouth along the aching tooth for the purpose of heating it. |
| *Osote /Gendela* | *Lactuca inermis* Forssk. | Asteraceae | Wh | Dr | Sexual weakness (Impotency) | After grinding the leaf into a powder and squeezing it, it is then combined with water. The drop of latex is collected, combined with powdered "teff," and baked before being consumed for the next three days before any other meal. |
| *Kosekoso* | Laggera tomentosa (A.Rich.) Sch.Bip. ex Oliv. & Hiern | Asteraceae | L | N, Dr | Tonsil, headache, fumigant in cleaning, leech in livestock | Leaf that has been ground up is squeezed and one cup of the juice is consumed for tonsil, headache and pain. The dried leaf portion is kept in the room to fumigate. |
| *Talba* | *Linum usitatissimum* L. | Linaceae | Se | Dr | Wound | The seed, after being soaked in water and cooked, cooled, and then consumed for wound and ulcer treatment. |
| *Kaseegammojji* | *Lippia abyssinica* (Otto & A.Dietr.) Cufod. | *Verbenaceae* | L | Dr, O | Herpes simplex virus | Dry leaves are chewed and swallowed and mixed with water and consumed. |
| *Wazimma* | *Medicago sativa* L. | Fabaceae | Fr | O, N | Tinea versicolor | To remove fungal infection on the skin, flower bund is applied as paste on the skin. |
| Birbira | *Millettia ferruginea* (Hochst.) Hochst . ex Baker | Fabaceae | L,Se,Fr, | Dr | Toothaches (L), wound (Se), earache(Fr), insecticidal (Se) | Leaves, seeds, and fruits are crushed and powdered before being dissolved in water. Adults consume one glass of the extract every day for five days, while children consume one cup of the extract every day for three days. This helps kill insects, and adults can also apply the powder to their ears by mixing it with water to treat earaches, and children can chew on the leaf to relieve toothaches for three days. |
| *Kataba* | *Myrica salicifolia* Hochst.ex A.Rich. | Myricaceae | Rb,R | O | Gonorrhea(R) and edema leg swelling (Rb) | Root and leaf are ground up, and water is extracted. After boiling and making a decoction of the entire plant with butter, one "kubaya" is consumed daily for five days in order to flush the system, and one cup is consumed daily in order to treat sexually transmitted diseases. |
| *Qacamoo* | *Myrsine africana* L. | Primulaceae | Fr | O | Dewormers (Kosso) | The fruit is soaked in water for a day, then consumed it thereafter. |
| *Shinet* | *Myrsine melanophloeos* (L.) R.Br. ex Sweet | Primulaceae | Lb,Fr | Dr, O | Diabetes mellitus (Lb), Scabies(Fr) | For diabetes, the plant parts are powdered and mixed with water and drunk 1 cup per day for five days; for scabies, paste and fruit combined powder is applied on the skin. |
| *Woyra* | *Olea europaea* L. | Oleaceae | L | Dr, E | Eye disease and fungus | Mixing grinding with water and applying it as a paste to the eye for an hour, then removing it and cleaning the area with tap water. and paste the mixture on the fungi infected area. |
| *Tifie* | *Olinia rochetiana* A.Juss. | Penaeaceae | L | Dr | Wound | Crushing the leaf, then combining it with water, and letting the mixture sit for an hour before applying it to wounds. |
| Tosign | *Origanum vulgare* L. | Lamiaceae | L | Dr,O | Skins Sores, aching muscles, asthma; cramping, diarrhoea | A glass of aqueous extract of the leaves to be consumed twice daily for diarrhoea. Rub the leaf powder on skin sores and hurting muscles. Drink a cup of the water extract of the leaves for five days to cure asthma. Roasted leaves should be ground, and the flour should be soaked in hot water; adequate salt should be added and the paste to be applied on cramps. |
| *Yelmachew* | *Oxalis corniculata* L. | Oxalidaceae | R,L | O,V | Vomiting (R&L) | Roots and leaves are ground up, diluted in water, and one "kubaya" is consumed daily for two days. |
| *Erbaa* | *Phyllopentas schimperi* (Hochst.) Y.D.Zhou & Q.F.Wang | Rubiaceae | L | O | General weakness | One cup of the juice made from powdered leaves and crushed leaves is consumed daily. |
| *Qundo arege* | *Periploca linearifolia* Quart.-Dill.&A.Rich. | Apocynaceae | Wh | Dr, O | Heart disease and wound | Crushed, diluted in water, and one can is consumed once a day for heart disease while the whole plant is chewed and then applied to wounds. |
| *Darguu* | *Phoenix sylvestris* (L.) Roxb. | Arecaceae | R,L | V | Diarrhoea with the presence of blood and mucus (L), hemorrhage (R), digestive(L), decreased passing of urine(R),.facilitate evacuation of the bowels(L), manage weight (L), Asthma  (L)  and, diarrhoea (L),  Hemorrhoids(R), rheumatic pains(R), itches and skin eruption(R,L). | Drinking one cup of juice prepared from the root and leaves of the plant is used for the treatment of all internal system disorders. For blood diarrhoea, one cup of crushed leaves and one cup of juice from the leaves are administered. Powdered root mixed with waste are applied to bleeding wounds. |
| *Handode* | *Phytolacca dodecandra* L'Hér. | Phytolaccaceae | R,L,Fr | O | Benign growth on the skin(L), (Warts), gonorrhea (R) and gastritis (Fr) | Crushed together with the shoot tip of *Cordia africana* Lam., the combination is salted, and one bottle of it is consumed daily for a week to treat gonorrhea and other sexually transmitted diseases.  After pricking the leaves, roots, and flowers with a gloved hand, cutting the leaves and spreading them out between two hides on the ground, and rubbing the ground to remove the leaves' stinging barbs so that they don't cause a burning sensation, the leaves can then be boiled, ground into a smooth paste, salted, and used to treat gastritis. |
| *Ganoxxobbi* | *Plantago lanceolata* L. | Plantaginaceae | L,St,R | Dr, O | Gastritis and benign growth on the skin  (Wart )(St,R) & trachoma(L) | Crushed and the concentrate juice is poured into the eye to treat eye discomfort. The leaf is crushed and a wet concentrate solution is applied to the wound to eliminate warts. The stem bark is cooked with kocho or enset juice and one "kubaya" is consumed to treat gastritis. |
| *Hoomi ,Burayya* | *Prunus zippeliana* Miq. | Rosaceae | Sb | O | Sexual weakness  (Impotence), tumor (hard swelling) | Crushed together with the bark of *Schefflera abyssinica* (Hochst. ex A.Rich.) Harms and cooked. One cup consumed once a week for three weeks for sexual weakness, and the powder is passed on by forceful swallowing. |
| *Gesho* | *Rhamnus prinoides* L'Hér. | Rhamnaceae | Fr | Dr, O | For pigment lost from areas of the skin, causing whitish patches (Vitiligo) | The flower is powdered and mixed with oil or butter and applied on the skin. |
| *Qadiida* | *Rhamnus staddo* A.Rich*.* | Rhamnaceae | R | O | The condition that causes you to wake up during the night to urinate (Nocturia) | Root is chewed and swallowed  during sleeping time |
| *Qobbo* | *Ricinus communis* L. | Euphorbiaceae | Fr | Dr | Fungal & infection during illegal abortion | One cup of the flower bund mixture, which has been crushed and mixed with water, is consumed daily for a week. |
| *Incibirri* | *Rubia cordifolia* L. | Rubiaceae | R,L | Dr | Hard swelling  (Tumor) | Root powder combined with water, or the juice of cooked leaves consumed one cup once a week . |
| *Amoch* | *Rubus steudneri* Schweinf. | Rosaceae | L | O | Liver disease (Hepatitis) | During the course of one month, one cup of leaf juice is consumed daily. |
| *Tsenadam* | *Ruta chalepensis* L. | Rutaceae | L,FI | O | Frequent, prolonged, and intense crying or fussiness in a healthy infant (Colicky babies) (F), diarrhoea(L), earache(L), heart pain(L), hemorrhoids (FI), influenza symptoms (FI) and intestinal disorders(L) | Small leaf is smoked in the room to cure colicky newborns, and the juice of the flower bud is taken one cup at a time, once daily, for two weeks to treat diarrhoea,. ear ache, heart discomfort, hemorrhoids and influeza. |
| *Maqimaqqo* | *Rumex abyssinicus* Jacq. | Polygonaceae | Wh | Dr, O | A small, hard, benign growth on the skin  (Wart) and sexual weakness (Impotency) | The juice of the entire plant is consumed for a period of one week for sexual weakness. The crushed whole plant is applied onto the diseased skin on a regular basis. |
| *Tult* | *Rumex nepalensis* Spreng. | Polygonaceae | R | O | A small, hard, benign growth on the skin  (Wart) & a dark patch of infected skin ( Tinea nigra) | In order to treat the skin, a solution of the root is made in oil is applied to it. |
| *Haye* | *Salix mucronata* Thunb. | Salicaceae | Rb | O | Disease caused of dogs (Rabies) | The disease is treated with root bud juice at a dose of one cup per day. |
| *Hulegeb* | *Salvia nilotica* Juss. ex Jacq. | Lamiaceae | Wh | N, O | Sleep-inducing effect  (Sedative), herpes simplex | The crushed solution is spread over hands and face. |
| *Garda/chifrig* | *Sida schimperiana* Hochst. ex A.Rich. | Malvaceae | L | Dr | Wound | The leaves either ground or powdered are applied on the wounds. |
| *Maera* | *Spiniluma oxyacantha* (Baill.) Aubrév. | Sapotceae | R | Dr | Wound | The injury is treated using a mixture of ground crushed roots. |
| *Ashkla harege* | *Smilax anceps* Willd. | Smilacaceae | R | Ha, O | Lung disease (Tuberculosis) | One cup of the juice extracted from the root once every seven days and consumed. |
|  | *Solanecio gigas* (Vatke) C.Jeffrey | Asteraceae | Wh | Dr | Sexual weakness (Impotency) | In order to cure sexual dysfunction, the herb is ground up and consumed in the shape of meal. |
| *Geber emboye/nechi/* | *Solanum marginatum* L.f. | Solanaceae | FR,St | O, N | Birth control & Sexual weakness (Impotence) (Fr &St), | Cooking flower buds under steam results in ma juice, which is used for both sexual weakness and birth control. |
| *Wulkifa* | *Sparrmannia ricinocarpa*  (Eckl. & Zeyh.) Kuntze | Malvaceae | R | O | Hepatitis | In order to cure hepatitis, root juice is administered once day in a cup along with tea. |
| *Enguchite /kalaala* | *Stephania abyssinica* (Quart.-Dill. & A.Rich.) Walp. | Menispermiaceae | Wh | Dr, O | General weakness, heart disease, cholera,  skin | The plant is processed into a kind of food that is cooked and consumed on a regular basis. |
| Yahya shito | *Tagetes minuta* L*.* | Asteraceae | L | O | Remedy for colds, respiratory, inflammations, stomach, problem, malaria, anti-parasitic, antiseptic, insecticide and sedative. | Smoked leaf is used for the treatment of malaria, and the juice of the plant is used daily for the treatment of respiratory diseases. |
| *Sirabizu* | *Thalictrum alpinum* L. | Ranunculaceae | Wh | N, O | Stomach pain | During periods of stabbing agony, the whole plant is ground up into a meal and consumed. |
| *Dobbi/samma* | *Urtica simensis* Hochst. ex A.Rich. | Urticaceae | L | O | Gastritis and sexual weakness | The juice of the plant is used for both gustatory and sexual purposes, but one should not consume more than one cup of it every day. |
| *Gurra harre/ketetina* | *Verbascum sinaiticum* Benth. | Scrophulariaceae | R,L | O, N | TB (R), Stomach(l) trouble(L), Wound(L), Syphilis (R) and heart failure infusion(L) | The powder is used to treat wounds, the leaf and root are ground up and made into a meal to treat sexually transmitted diseases. One cup of the juice is consumed to treat heart and stomach conditions. |
| *Dheebicha/ girawa* | *Gymnanthemum amygdalinum* (Delile) Sch.Bip. | Asteraceae | L | Dr, O | Toxicity and wound, leech from livestock | To treat toxicity, it is necessary to remove wounds that extend beyond the plant's leaves and consume juice. |
| *Gujo /reji* | ***Orbivestus leopoldii* (Sch.Bip. ex Walp.) H.Rob..** | Asteraceae | R,L | Dr, N | Snake bite (L) and menstruation problem (R) | Crushed, chewed, or powder combined with water is consumed as juice for treating snake bite and reducing the discomfort associated with menstruation. |
| *Araressa/harege eressa* | *Zehneria scabra* subsp. *scabra* | Cucurbitaceae | L,St | Dr,N,O | Gout arthritis (L), wart(St) | It is recommended that patients eat one cup of juice made from cooked steam and leaves every day for one month in order to get rid of gout and arthritis, and that stem powder is applied to warts. |

**Abbreviations:** Both (B), Chewing (Ch), Climber (Cl), Concoction (Co), Crushing (Cr), Crushing and homogenizing with water (Chw), Dermal (Dr), Dried (D), Eye ointment (Eo), Flower (Fl), Flower bud (Fb), Fruit (Fr), Fresh (F), Hanging (Ha), Head (Hd), Herb (H), Infusion (In), Internal fiber (Fi), Leaves (L), Leaves bud (Lb), Lianas (Li), Life form (LF), Methods of preparation and application (MPAP), Nasal (N), Oral (O), Plant part used (PU), Pounding (P), Powdering (Pw), Relative Frequency of Citations (RFC), Route of administration (RA), Root (R), Root bark (Rb), Seed (Se), Shrub (S), Smoke bath (Smb), Squeezing (Sq), Stem (St), Stem bark (Sb), Steam bath (Stb), Tree/shrub (TS), Tree (T), Vaginal (V), Vernacular Name (VN), Whole (Wh)

The rows highlighted in yellows involve the preparation of medicine by mixing two or more medicinal plants.


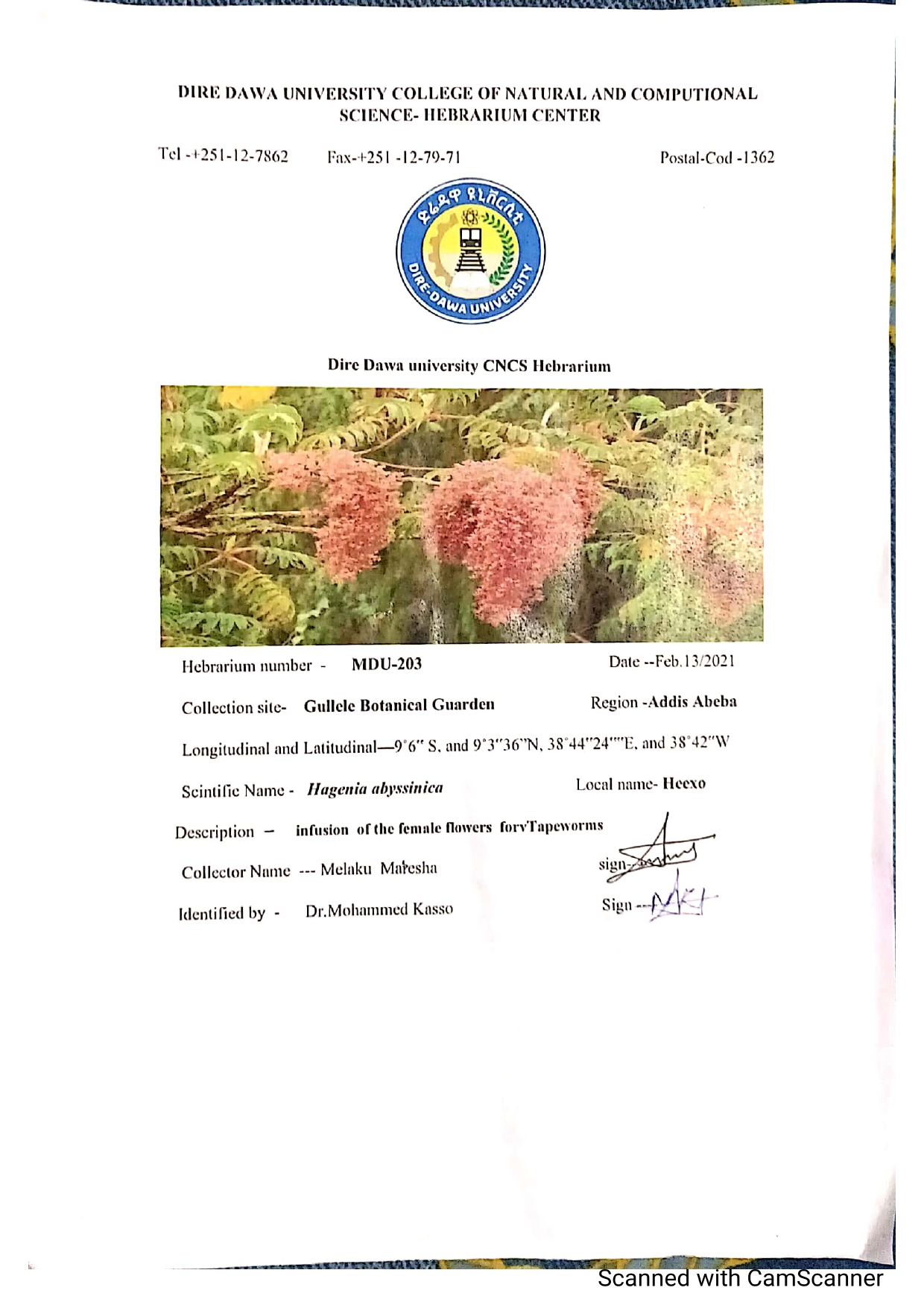


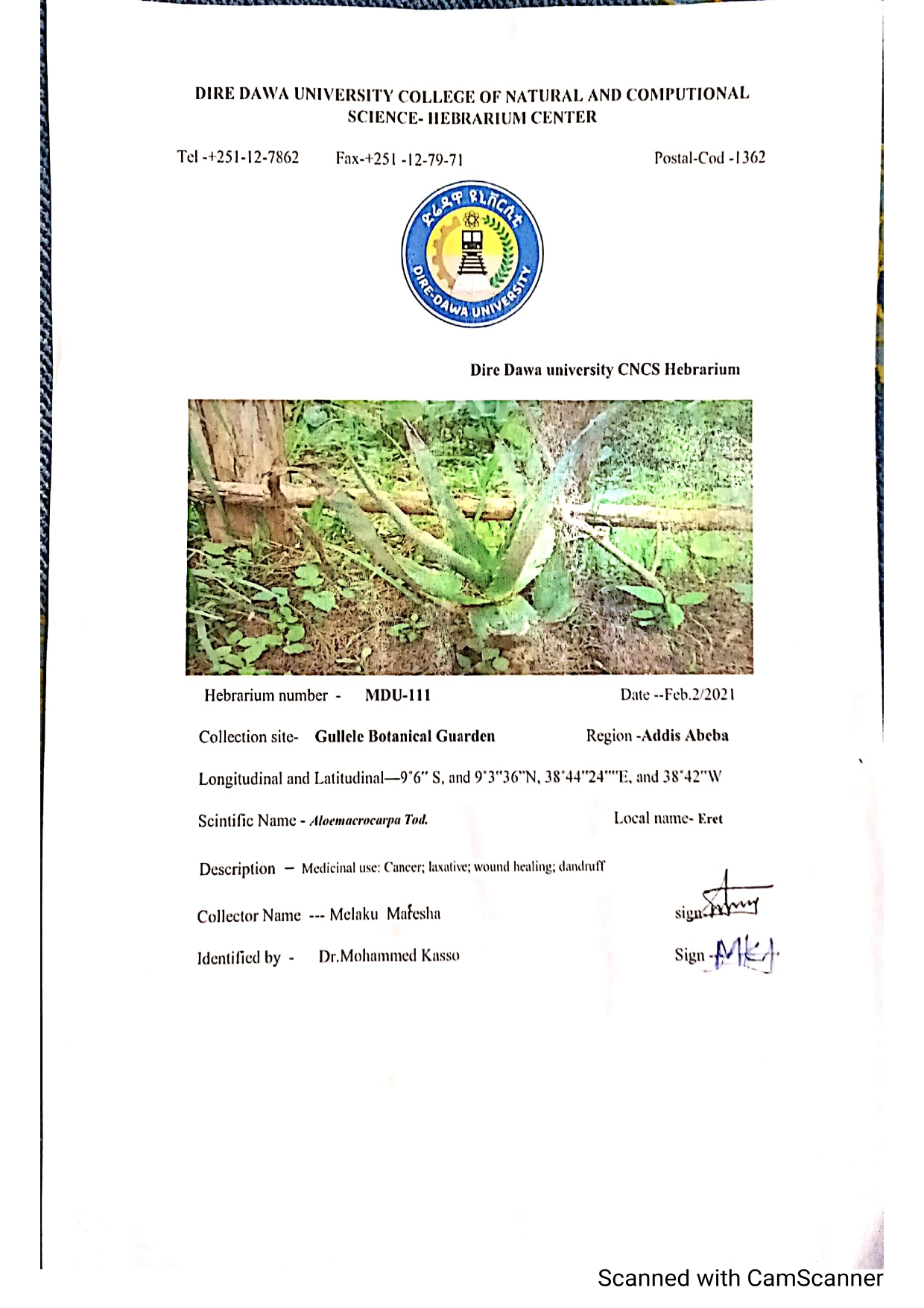


**Supplimentary -6 - Some formulation and of Medecinal plants**


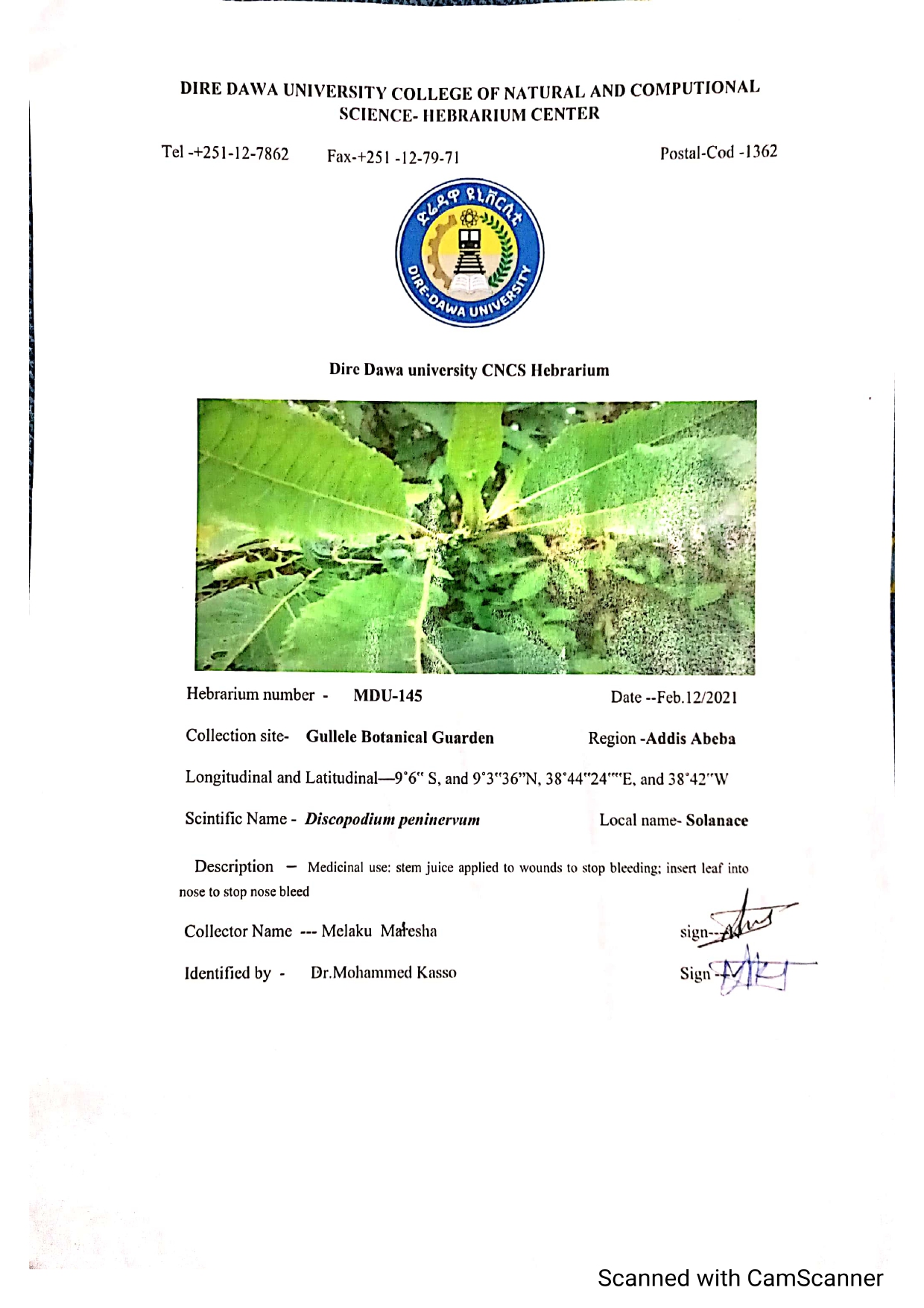

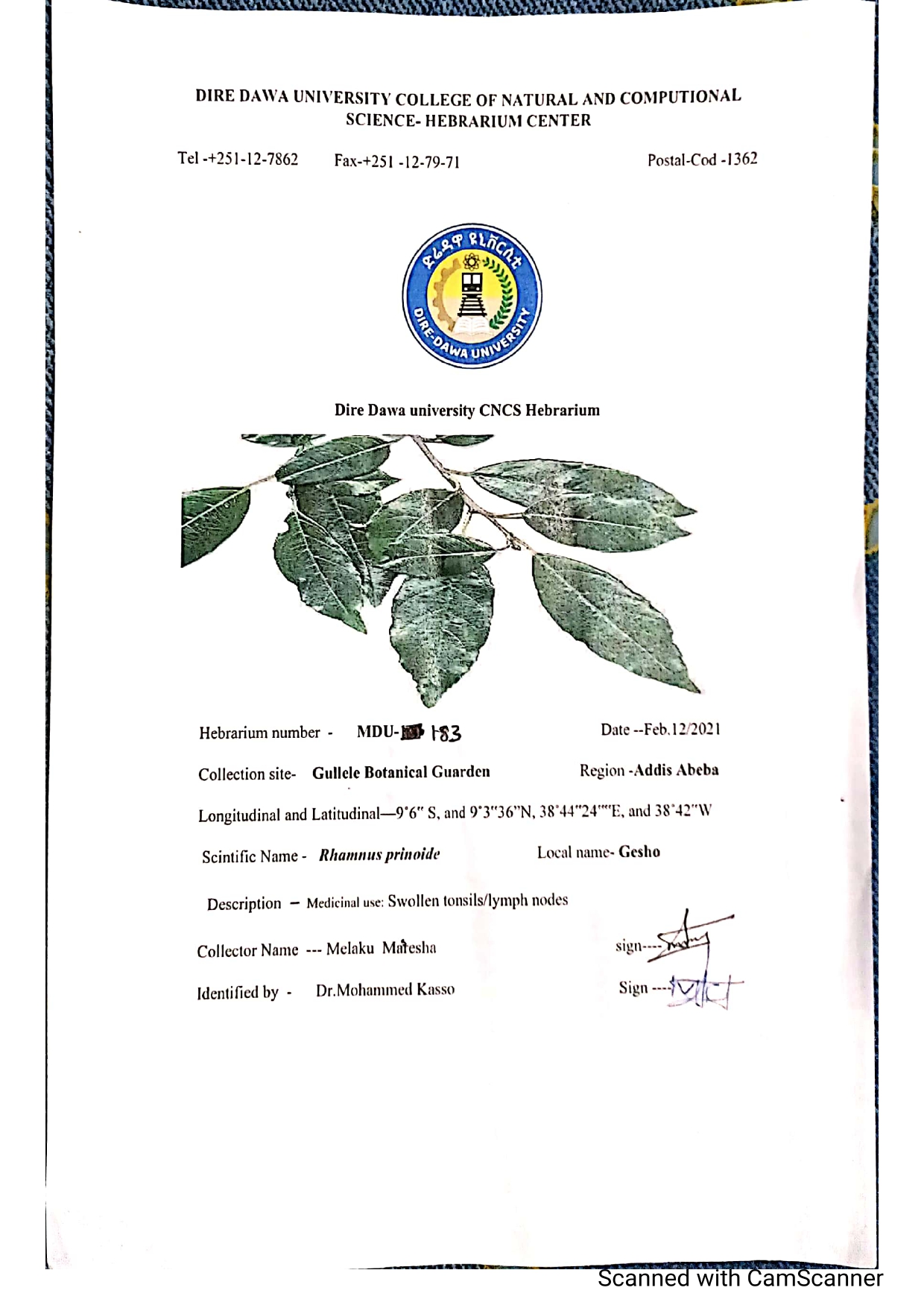


Figure S1 Voucher specimens of some of the collected medicinal plants submitted to the herbarium of Dire Dawa University, Ethiopia.


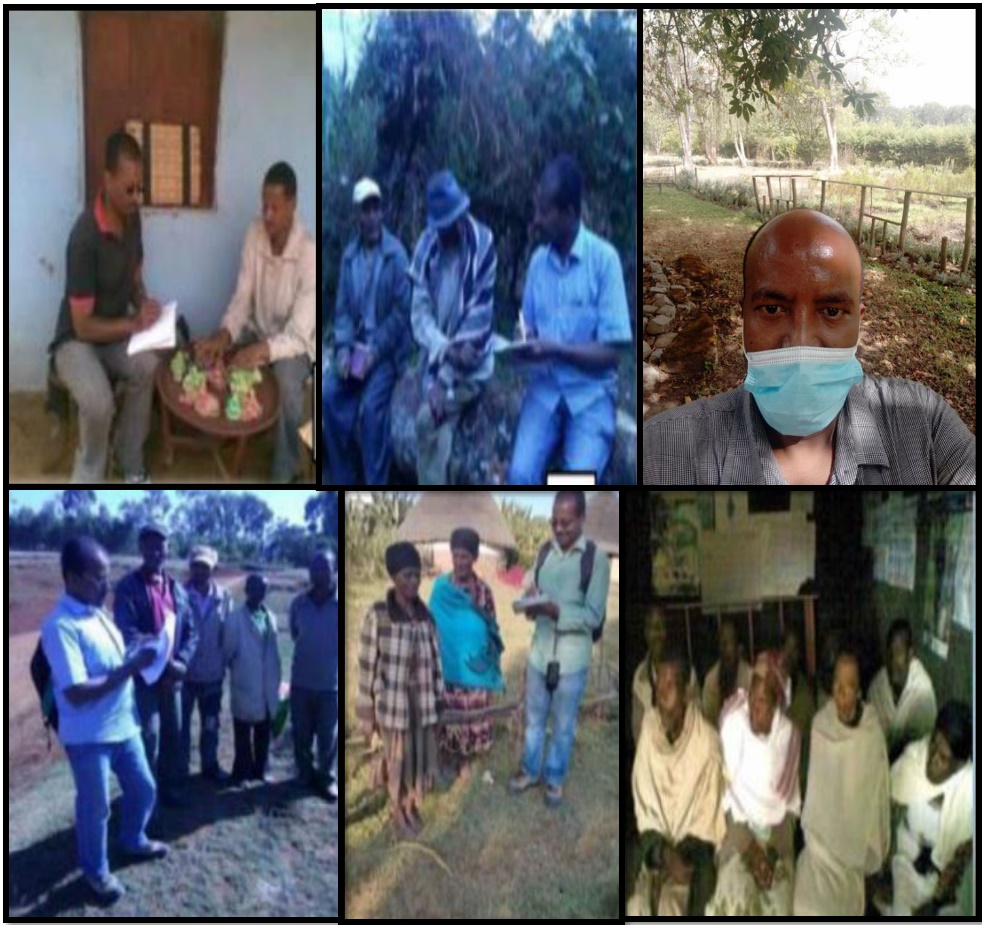


Figure S2 Representative picture of Dr. Mohammed Kasso Geda interviewing the traditional healers in Gullele Botanical Garden.
